# Therapeutic potential of nitric oxide and its donors in hemorrhagic and ischemic stroke: a systematic review

**DOI:** 10.4103/mgr.MEDGASRES-D-25-00161

**Published:** 2026-01-06

**Authors:** Shijun Wang, Sicen Wan, Xu Zhang, Ming Sun, Hongru Li, Gang Chen, Jiahe Wang, Xiang Li

**Affiliations:** Department of Neurosurgery & Brain and Nerve Research Laboratory, The First Affiliated Hospital of Soochow University, Suzhou, Jiangsu Province, China

**Keywords:** cerebral blood flow, cerebral vasospasm, intracerebral hemorrhage, ischemic stroke, neuroinflammation, neurotransmission, nitric oxide, nitric oxide synthase, subarachnoid hemorrhage, treatment strategy

## Abstract

**Facts**

1. Nitric oxide (NO) exhibits dual roles in stroke pathophysiology, serving as both a neuroprotective agent through vasodilation and anti-thrombotic effects, and a neurotoxic mediator via oxidative stress and glutamate excitotoxicity, depending on its concentration, timing, and source.
2. NO has been shown to play a critical role in improving outcomes after stroke in experimental studies.
3. The efficacy of NO-based interventions varies depending on stroke type and timing. The administration of NO and its donors in stroke therapy should be flexibly integrated based on the temporal dynamics of endogenous NO following stroke onset. Inappropriate timing of medication may exacerbate the patient’s condition post-stroke.

**Open questions**

1. What are the precise molecular mechanisms underlying the concentration-dependent dual effects of NO in different stroke subtypes, and how can we selectively enhance its beneficial while suppressing its detrimental actions?
2. How can we optimize the timing and dosing of NO donors across heterogeneous stroke populations, especially in intracerebral hemorrhage where clinical evidence is notably scarce?
3. Can NO-based interventions be safely translated from animal models to human patients? The limited number of clinical studies necessitates larger-scale trials to confirm efficacy and safety in diverse patient populations.
4. What are the long-term neurological consequences of NO administration? While short-term benefits have been observed, the potential for delayed adverse effects or unintended impacts on brain function remains underexplored.

Nitric oxide is a gas molecule that serves as a signaling molecule in mammals, regulating the relaxation and contraction of vascular, thereby modulating local blood flow. Stroke encompasses both hemorrhagic and ischemic subtypes, with hemorrhagic strokes further classified into subarachnoid and intracerebral hemorrhages. The vasodilatory effects of nitric oxide and its derivatives have been confirmed in both peripheral and central vascular diseases. Additionally, animal studies have demonstrated that exogenous supplementation of nitric oxide or its donors has beneficial effects on stroke. We systematically reviewed the existing research on the relationship between nitric oxide donors and stroke, and elaborated on the pathophysiological processes in which nitric oxide is involved in different types of strokes. Given the significant differences in the concentration and temporal effects of nitric oxide in various types of strokes and their pathophysiological processes, the optimal timing for exogenous nitric oxide intervention under different conditions was analyzed to enhance clinical awareness regarding the treatment with nitric oxide and its donors. Future research can place greater emphasis on the development of novel nitric oxide donors and their diverse administration routes to further optimize treatment outcomes for stroke. This review underscores the limited progress in the clinical translation of nitric oxide-based therapeutics and aims to offer novel insights for the further optimization of their development, delivery strategies, and administration timing.

## Introduction

In 1772, nitric oxide (NO) was first discovered in the external environment. It was not until the 1990s that scientists discovered that NO functions as a signaling molecule in mammals, playing a role in vasodilation.^1^ NO is a lipophilic gas molecule composed of one nitrogen atom and one oxygen atom, exhibiting high reactivity, which results in its short-lived presence in the body, often existing in the form of nitrates and nitrites. Previous studies have confirmed that NO plays critical roles in vascular smooth muscle relaxation, inhibition of platelet aggregation, and anti-inflammatory processes.^2^ Based on these physiological functions, numerous drugs have been developed for clinical use. For instance, sublingual nitroglycerin effectively dilates coronary arteries and alleviates angina by promoting NO production in the vascular endothelium.^3^ However, as a strong oxidant, NO can also participate in oxidative reactions within the body. Under pathological conditions, excessive NO production can lead to the generation of nitrogen-containing free radicals, triggering inflammatory responses, circulatory shock, and ischemia–reperfusion injury.^4^

Stroke is one of the most common cerebrovascular accidents today, encompassing subarachnoid hemorrhage (SAH), intracerebral hemorrhage (ICH), and ischemic stroke (IS). According to the latest Global Burden of Disease report, stroke ranks third among the leading causes of adult mortality, with 11.9 million new cases reported in 2021 and stroke-related deaths rising to 7.25 million.^5^ Major risk factors for stroke include hypertension, diabetes, dyslipidemia, poor dietary and lifestyle habits, psychological factors, and certain genetic predispositions. Additionally, environmental factors, particularly air pollution, are considered to have a notable association with stroke incidence.^6^ Given that NO is a common air pollutant and an important signaling molecule in the body, its role in stroke has garnered our attention.

SAH is a specific type of brain hemorrhage characterized by blood from a ruptured vessel entering a distinct space, the subarachnoid space. The metabolites in the blood entering the subarachnoid space, such as hemoglobin (Hb), along with the accompanying inflammatory response, stimulate surrounding blood vessels, leading to smooth muscle contraction—a phenomenon known as cerebral vasospasm (CVS).^7^,^8^ Following CVS, patients may experience corresponding ischemic symptoms in brain tissue, potentially resulting in severe ischemic complications.^9^ However, as clinical observations progressed, researchers discovered that not all patients experiencing CVS exhibited symptoms of brain ischemia.^10^ As a result, studies exploring the relationship between NO and SAH have been relatively limited in recent years. Delayed cerebral injury is closely linked to various pathophysiological changes, such as microcirculatory disturbances, blood–brain barrier (BBB) dysfunction, and impaired autoregulation of cerebral blood vessels, with NO playing a crucial role as a key signaling molecule in these processes.^11^

ICH refers to the rupture of blood vessels within the skull, leading to the leakage of blood into the brain parenchyma and resulting in tissue damage. The most common type of ICH is hypertensive hemorrhage. Blood released from the ruptured vessels provides hypoxic red blood cells to the brain tissue, and the subsequent rupture of these cells and the deposition of hemosiderin can further trigger neuroinflammation, leading to neuronal damage.^12^ Additionally, the formation of blood clots is closely related to platelet aggregation, a process in which NO is also involved. The rupture of red blood cells releases Hb, and there is a strong connection between Hb in a hypoxic state and the local production of NO, contributing to the neuroinflammatory response following a brain hemorrhage.^13^ Furthermore, the association between NO and hypertension indirectly affects the incidence of ICH.^14^

IS is characterized by the interruption of blood supply to localized brain tissue due to vascular occlusion.^15^ Patient prognosis is closely linked to the salvaging of the ischemic penumbra. Severe disability from IS often occurs when ischemic brain tissue cannot recover adequate perfusion within a short timeframe, leading to further tissue damage and loss of neurological function.^16^ Recombinant tissue plasminogen activator is the thrombolytic agent currently recommended by clinical guidelines for IS patients within a 4.5-hour onset window, aimed at minimizing the area of the ischemic penumbra and preserving neurological function.^17^,^18^ However, regardless of vascular recanalization, neurological damage caused by ischemia is inevitable. NO is involved in the occurrence of neuroinflammation following IS, the dysfunction of cerebral endothelial cells, neuronal death, and the disruption of the BBB following IS.^19^ Previous studies have also indicated that supplementing NO donors within the therapeutic time window may have a positive impact on the prognosis of IS patients.^20^,^21^,^22^

Approximately three decades ago, researchers had already identified a significant association between NO and cerebrovascular diseases. They initiated comprehensive and detailed investigations into NO donors and their production pathways, with a particular emphasis on the NO synthase (NOS) pathway. These pioneering studies not only clarified the essential role of NO in microvascular injury during ischemia-reperfusion but also established a foundation for subsequent clinical intervention strategies.^23^,^24^,^25^ Since 2010, there has been an increase in research efforts aimed at enhancing stroke outcomes through the modulation of the NOS pathway. This surge in research has allowed scientists to explore the complex roles of NO in the post-stroke environment more thoroughly. Numerous pharmacological interventions targeting the NOS pathway have been identified, elucidating the specific mechanisms by which NO influences stroke pathophysiology. Since 1994, researchers have been actively investigating both inorganic and organic NO donors. Until now, advancements in drug delivery methods and platforms have facilitated the direct delivery of NO across the BBB, overcoming a previously formidable obstacle. Despite these significant strides, a comprehensive synthesis of studies related to the direct delivery of NO or its precursors remains conspicuously absent.

This review systematically synthesizes evidence from both seminal historical studies and recent investigations over the past three decades to delineate the evolving roles of NO and its donors across three major stroke subtypes. By integrating foundational insights with contemporary findings, we aim to clarify the pathophysiological mechanisms and therapeutic potential of NO-based strategies in SAH, ICH, and IS. Furthermore, this work highlights persistent challenges and opportunities within the field, particularly the slow progress in clinical translation, and seeks to provide a balanced, critical foundation for future research aimed at optimizing timing, delivery, and subtype-specific application of NO donors to improve patient outcomes.

## Methods

### Search strategy

The systematic review was followed the Preferred Reporting Items for Systematic reviews and Meta-Analyses (PRISMA) guidelines.^26^ A comprehensive literature search was conducted across three primary databases: PubMed, ScienceDirect, and the Cochrane Library. The search used the terms “Nitric Oxide,” “SAH,” “ICH,” “IS” and “treatment.” The literature search was limited to the period from 1990 to the present. Detailed search strategies are provided in **Additional file 1**. Initial selection of articles was based on the relevance of their titles or abstracts to the search terms, followed by a de-duplication process across the databases. The remaining articles were imported into Endnote 20 (Clarivate, Philadelphia, PA, USA) for further de-duplication using software algorithms. Subsequently, these articles underwent a rigorous screening process, including title screening, abstract screening, and full-text review, to determine their eligibility for inclusion in this review.

### Eligibility criteria

For animal experiments, the inclusion criteria were as follows: (1) healthy mice, rabbits, or non-human primates in which SAH or cerebral infarction was induced by validated methods; (2) intervention with NO, a direct NO donor, or an NO precursor such as L-arginine (L-Arg) or nitrite; (3) drug administration temporally linked to the stroke event; and (4) outcomes pertinent to stroke prognosis. The exclusion criteria were endpoints unrelated to cerebrovascular or neurological recovery.

For clinical research, the inclusion criteria were as follows: (1) Individuals with a confirmed history of stroke, regardless of stroke subtype; (2) Administration of NO, a direct NO donor, or an NO precursor such as L-Arg or nitrite; (3) drug administration was timed in relation to stroke onset; (4) Studies reporting outcomes relevant to stroke prognosis; and (5) The included study types comprised intervention studies. Observational studies, case reports and review articles were excluded.

This review specifically concentrates on NO donors and associated pharmacological agents, thereby excluding studies examining the NOS pathway that might impact stroke outcomes.

### Study selection and data collection

All articles and reference lists of the randomized controlled trials (RCTs) and non-RCTs from the systematic search in the electronic database were evaluated separately in accordance with previously mentioned the eligibility criteria. After the rigorous assessment and evaluation of the literature by two different reviewers (SWang and SWan), the data were extracted from the included studies: literal information such as author and publication year, inclusion and exclusion criteria, treatment method and cerebral vascular outcomes.

### Risk of bias assessment

Two reviewers (SWang and SWan) independently assessed the risk of bias of included studies using the Cochrane Risk of Bias Tool (RoB 2.0, Cochrane, Oxford, UK) for RCTs,^27^ the Risk of Bias in Non-randomised Studies of Interventions tool (ROBINS-I, Cochrane) for non-RCTs^28^ and the SYstematic Review Centre for Laboratory animal Experimentation (SYRCLE)’s Rob Tool (Radboud University Medical Center, Nijmegen, the Netherlands) for animal experiments.^29^ Discrepancies were resolved through consensus or consultation with a third reviewer (XZ). This systematic approach ensured an objective and transparent evaluation of potential biases in the included studies.

### Risk of Bias Analysis of Included Studies

**Figure 1:**
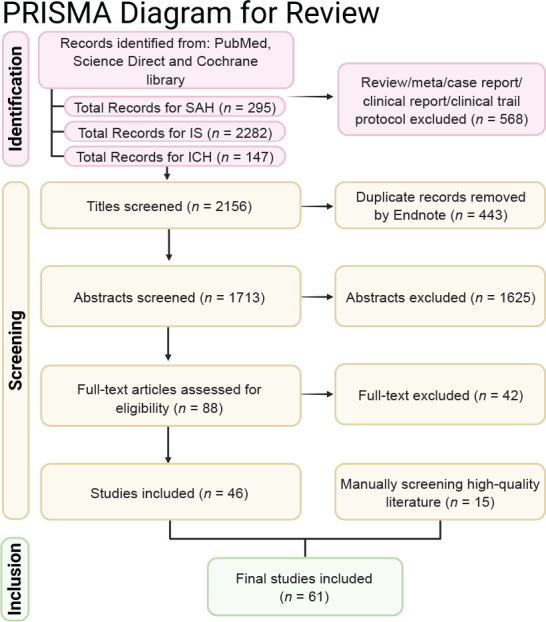
The PRISMA flow diagram for NO and SAH, ICH and SAH. Created with BioRender.com. ICH: Intracerebral hemorrhage; IS: ischemia stroke; NO: nitric oxide; PRISMA: Preferred Reporting Items for Systematic reviews and Meta-Analyses; SAH: subarachnoid hemorrhage.

This research synthesis incorporated a total of 61 experimental studies, categorized by pathology and model type. SAH was investigated in 27 studies, comprising 25 preclinical animal studies and two human clinical studies, both of which were prospective RCTs. Regarding IS, 33 studies were included: 25 utilized animal models, while eight were clinical investigations. Among these clinical IS studies, seven were designed as RCTs and one was a prospective non-RCT. Finally, ICH was represented by a single clinical study, conducted as an RCT. Detailed information is systematically presented in **Tables 1–4**. All databases were searched following the guidelines of the PRISMA, with the consolidated PRISMA flow diagram was shown in **Figure 1**.

**Table 1:** Animal experiments of nitric oxide (NO) in subarachnoid hemorrhage

Study	Year	Animal	Source of NO	Route of administration	Dosage of source of NO	Time to giving source	Control group	Results
Pluta et al.^69^	1997	Cynomolgus monkeys	NONOates	Acute intravenous infusion Long-term intravenous infusion	10 μM 1 μM	7 d after SAH Started 24 h after SAH induction and continued for 7 d	Saline-infused group	Reversal of delayed cerebral vasospasm with acute intracarotid infusion of an NO donor
Wolf et al.^70^	1998	Canine	DETA/NO	Intrathecal bolus via the cisterna magna	0.2 μmol 2 μmol	7 d after SAH	2 μmol of the inactive drug carrier DETA group	Intrathecal DETA/NO effectively reverses vasospasm
Kiris et al.^71^	1999	New Zealand White rabbits	SNAP	Intravenous infusion	15 mg/kg/min	46 h after SAH	Saline-infusion gruop	NO donor SNAP is a potentially useful drug to reverse cerebral vasospasm due to SAH
Pluta et al.^72^	2000	Cynomolgus monkeys	L-arginine	Acute intravenous infusion Long-term intravenous infusion	1 × 10^-6^ M 1 × 10^-3^ M	7 d after SAH Started 24 h after SAH induction and continued for 7 d	Sham and saline-infusion groups	L-arginine increased regional cerebral blood flow
Sun et al.^73^	2003	Rats	L-arginine	Intraperitoneal injection	0.5 g/kg/body weight	30 min before SAH and every 6 h for 24 h post-SAH	Sham and saline-infusion groups	L-arginine improves microcirculatory perfusion and provides a protective effect against secondary cerebral ischemic injury
Göksel et al.^74^	2001	Rabbits	L-arginine	Continuous intracisternal infusion	300 mmol	4 d after SAH	Sham and saline-infusion groups	Continuous intracisternal L-arginine infusion effectively resolved cerebral vasospasm in the SAH model
Sönmez et al.^75^	2002	New Zealand White rabbits	SPER/NO	Intracarotid arterial infusion	0.33 mL/kg (325.2 mmol/mL)	24 h after SAH	Sham and saline-infusion groups	The SPER/NO treatment effectively alleviated post-SAH vasospasm
Gabikian et al.^76^	2002	Rabbits	20% DETA/NO-EVAc	Implantation	0.48 mg/kg	30 min after SAH	Blank polymers implantation group	DETA/NO-EVAc polymer significantly prevented vasospasm
Pradilla et al.^77^	2004	Rabbits	20% DETA/NO-EVAc	Implantation	0.5 mg/kg 1.3 mg/kg	24 h after SAH 48 h after SAH 24 h after SAH 48 h after SAH	Empty EVAc polymers group	No significant effect The lumen patency of the basilar artery is significantly increased
Clatterbuck et al.^81^	2005	Cynomolgus monkeys	20% DETA/NO-EVAc	Implantation	4.3 mg/kg	After SAH	Blank polymers implantation group	The controlled release of DETA/NO effectively prevents delayed cerebral vasospasm
Aihara et al.^82^	2003	Monkeys	DETA/NO	Continuous intracisternal infusion	12 mL/d (1 mM)	After SAH and continued for 7 d	DETA treatment group	DERA/NO has no preventive effect on vasospasm
Lambert et al.^78^	2001	Wistar rats	SNP	Intravenous infusion	18 μg/h	3 h after SAH	Bosentan and no treatment groups	SNP can effectively reverse sympathetic nerve excitation after SAH, improve vasospasm, and promote the recovery of cerebral blood flow
Macdonald et al.^79^	2002	Monkeys	SNP	Continuous intracisternal infusion	11.2 mg total	After SAH and continued for 7 d	5% glucose infusion gruop	High-dose sodium nitroprusside did not prevent vasospasm in the primate SAH model
Lilla et al.^80^	2016	Male Sprague-Dawley rats	SNP	Intravenous infusion	1.5 μg/kg/min	15 min after SAH induction and continued for 180 min	2.5mL purified water in 5% glucose infusion gruop	Low-dose treatment with SNP can attenuate early perfusion deficits following SAH and reduce neuronal damage, despite inducing hypotension
Li et al.^83^	2013	Male Swiss Webster mice	NO	Breathing machine	60 ppm for 8 h	After SAH	20 and 80 ppm inhalation groups	iNO (60 ppm for 8 hours) improved recovery in SAH without worsening bleeding and reduces the inflammatory response
Terpolilli et al.^84^	2016	C57BL mice	NO	Breathing machine	50 ppm	3 h after SAH	Sham group	Inhalation of NO significantly reduced pial arteriolar spasms and reduced brain edema and hippocampal neuronal loss
Ito et al.^85^	2000	Rabbits	GTN	Tape (percutaneous)	0.675 mg	After SAH	Placebo or saline group	Low-dose nitroglycerin tape effectively improves cerebral vasospasm following SAH
Marbacher et al.^86^	2008	New Zealand White rabbits	GTN	Continuous intrathecal infusion	0.25 μg/h	After SAH continued for 5 d	Saline-infused group	GTN significantly prevented SAH-induced vasospasm
Ramdurg et al.^87^	2010	Wistar Abino rats	GTN	Intracisternal infusion	0.1 mg/kg	2 h after SAH	Papaverine gruop	Short-acting NO donors are less effective at alleviating vasospasm
Fathi et al.^88^	2011	Rabbits	GTN	Continuous intrathecal infusion	0.25 mg/h	After SAH continued for 5 d	Saline-infused group	GTN significantly prevented vasospasm in the basilar artery
Pluta et al.^89^	2005	Cynomolgus monkeys	NaNO_2_	Intravenous infusion	90 mg for 24 h + 45 mg/d (bolus) 180 mg for 24 h	1 h After SAH	Saline-infused group	Subacute NaNO_2_ infusions prevented delayed cerebral vasospasm; Inverse relationship between CSF nitrite levels and vasospasm severity
Fathi et al.^90^	2011	Cynomolgus monkeys	NaNO_2_	Intravenous infusion	300 μg/kg/h	7 d after SAH	Saline infusion group	Intravenous infusion of NaNO2 can reverse SAH-induced vasospasm in primates
Sehba et al.^91^	2007	Male Sprague-Dawley rats	GSNO	Intravenous infusion	1 μmol/8 μL/min	15 min after SAH	Saline-infused group	GSNO-treated SAH rats exhibit increased levels of collagen IV and EBA immunofluorescence, along with reduced collagenase activity
Afshar et al.^92^	1995	Cynomolgus monkeys	Saturated NO solution	Internal carotid artery infusion	1 mL/min for 3 min (1.3 mM)	7 d after SAH	Sham and saline-infusion groups	Intracarotid infusion of NO can reverse cerebral vasospasm
Huang et al.^93^	2014	Male Sprague-Dawley rats	NO-ELIP	Intravenous infusion	500 μg	30 min after SAH	Saline or non-NO-ELIP group	Ultrasound control of NO release from NO-ELIP can effectively improve vasospasm after SAH

CSF: Cerebrospinal fluid; DETA/NO: diethylenetriamine-NO; DETA/NO: diethylenetriamine-NO; EBA: endothelial barrier antigen; EVAc: ethylene-vinyl acetate; GSNO: S-nitroso glutathione; GTN: glyceryl trinitrate; iNO: inhaled NO; NaNO_2_: sodium nitrite; NO-ELIP: NO loaded-echogenic liposome; NONOates: NO-donating compounds; ppm: parts per million; SAH: subarachnoid hemorrhage; SNAP: S-nitroso-N-acetyl penicillamine; SNP: sodium nitroprusside; SPER/NO: spermine/nitric oxide complex.

**Table 2:** Clinical trials of nitric oxide (NO) in subarachnoid hemorrhage (SAH)

Study	Year	Study type	Disease	Source of NO	Route of administration	Dosage of source of NO	Time to giving source	Control gruop	Results
Reinert et al.^94^	2004	RCT	Patients suffer from SAH	GTN	Transdermal	14 μg/kg/h	1-9 d after SAH	Standard-treatment group	Transdermal nitrogylcerin positively influences cerebral blood flow
Ezra et al.^95^	2024	RCT	Patients with low grade aneurysmal SAH	Sodium nitrite	Intravenous infusion	10 μg/kg/min	Mean 66 h after SAH	Saline placebo infusion group	Intravenous sodium nitrite supplementation can effectively increase cerebral blood flow

GTN: Glyceryl trinitrate; RCT: randomized controlled trial.

**Table 3:** Animal experiments of nitric oxide (NO) in ischemic stroke

Study	Year	Animal	Source of NO	Route of administration	Dosage	Time to giving source	Control group	Results
Zhang et al.^108^	1994	Spontaneously hypertension rats	SNP SIN-1	Carotid artery infusion	3 mg/kg/h 1.5-6 mg/kg/h	3-5 min after MCAO	Saline infusion group	NO donor can effectively increase cerebral blood flow in the stroke area and reduced infarct size
Stagliano et al.^109^	1997	Wistar rats	SIN-1	Intra-arterial infusion	3 mg/kg	Immediately after the formation of thrombosis	Sham group	Improved sensorimotor function and hippocampal protection
			L-Arg	Intravenous injection	300 mg/kg			No significant effect
Ramos-Zuniga et al.^110^	1998	Wistar rats	Isosorbide dinitrate	Subcutaneous injection	5 mg/kg	1 h before MCAO, and repeat every 3 h	Saline injection group	Isosorbide dinitrate significantly preserved viable neurons
Pluta et al.^111^	2001	New Zealand White rabbits Sprague-Dawley rats	ProliNO	Intracarotid infusion	1 × 10^-6^ M 1 × 10^-5^ M	60 min after embolization Within 2 min after reperfusion	NaOH infusion group	ProliNO can improve the recovery of nerve function after reperfusion by reducing free radicals
Khan et al.^112^	2006	Sprague-Dawley rats	GSNO	Intravenous injection	2-3 μmol/kg	20 min after MCAO	Sham and stroke with saline injection groups	GSNO demonstrated the most potent cerebrovascular protection by reducing oxidative stress and inflammation
Prado et al.^122^	1996	Spontaneously hypertension rats	L-Arg	Intraperiton and intravenous injections	300 mg/kg	After MCAO	Sterile water injection group	No significant effect
Sadoshima et al.^123^	1997	Spontaneously hypertension rats	L-Arg	Intravenous injection	300 mg/kg	Before MCAO	Saline infusion group	L-Arg infusion can increase the preservation of glucose and ATP in ischemic brain tissue and the recovery of brain tissue after perfusion
Lapchak et al.^124^	2015	New Zealand White rabbits	tPA + L-Arg	Intravenous injection	3.5 g/100 mL	1 h after MCAO	Saline, saline with L-Arg, and tPA vehicle groups	L-Arg does not improve cerebral blood flow after stroke, but it does not adversely affect stroke outcomes
Martinez-Murillo et al.^113^	2007	Wistar rats	LA 419	Oral gavage	20 mg/kg	6 and 3 h before stroke onset	Vehicle group	LA 419 can effectively improve cerebral ischemic area and has cerebral protection
Serrano et al.^114^	2007	Wistar rats	LA 419	Oral gavage	20 mg/kg	1 h before stroke onset	Vehicle group	LA 419 can prevent the damage of brain nitrogen-energy system balance and improve I/R injury
Jung et al.^125^	2006	Sprague-Dawley rats	Sodium nitrite	Intravenous injection	48-4800 nmol	Begin with reperfusion after stroke onset	Saline-treated I/R group	48 and 480 nmol nitrite infusion can reduce infarct size
Mason et al.^126^	2000	Wistar rats	DEA/NO	Femoral venous injection	1 mL/100 g (1 × 10^-6^ M)	At the onset of reperfusion	Saline vehicle group	Infusion of NO donor can reduce the generation of free radicals in the ischemic region
Cui et al.^127^	2009	C57BL/6J mice	DETA/NONOate	Intravenous injection	0.4 mg/kg	24 h after MCAO	MCAO with PBS injection group	DETA/NONOate can promote the migration of neuroblasts after stroke
Chen et al.^128^	2004	Wistar rats	DETA/NONOates & hMSCs	Intravenous injection	0.4 mg/kg DETA/NONOates + 1 × 10^6^ hMSCs	24 h after MCAO	PBS injection group	NO donor combined with hMSCs can promote angiogenesis and neurogenesis after stroke
Greco et al.^115^	2007	Male Wistar rats	GTN	Systemic administration	10 mg/kg	20 min before MCAO	PEG injection group	The cerebral infarction area pretreated with GTN was reduced; the neuroprotective effect of GTN was limited.
Maniskas et al.^116^	2019	Male C57BL/6 mice	GTN	Intra-arterial injection	3.12-25 μg/μL	5 min after MCAO	Vehicle injection group	GTN has a certain neuroprotective effect after stroke, especially at the dose of 12.5 μg/μL
Sorby-Adams et al.^117^	2021	Purebred female merino sheep	GTN	Transdermal/subdermal injection	0.2 mg/h	1 h after MCAO	Sham group and placebo group	GTN can reduce the size of cerebral infarction, relieve cerebral edema and reduce intracranial pressure
		Male C57BL/6 mice			Varied doses	20 min after MCAO	Sham group and PEG injection group	No significant effect
Li et al.^118^	2020	Male C57BL/6 mice	PAMNs + L-Arg	Intravenous injection	200 μL	After stroke onset	Sham/stroke blank/free L-arginine group	PAMNs can be rapidly located at the site of ischemic injury and directly supplement L-arginine
Zhang et al.^119^	2023	Male Sprague-Dawley rats	PEI-PO-NONOates	Nasal administration	0.13 μmol/kg	After stroke onset	Sham group and MCAO with saline nasal administration group	Nasal administration of NO donors can reduce brain infarct volume
Zhuang et al.^129^	2010	Sprague-Dawley rats	ZJM-289	Intraperitoneal injection	0.1 and 0.2 mmol/kg	1 h before stroke onset	Sham and DMSO-saline groups	ZJM-289 can reduce infarct size and promote neurological function recovery
Atochin et al.^130^	2016	C57BL6/J mice	IQ-1S	Intraperitoneal injection	25 mg/kg	30 min before MCAO and 24 h after stroke onset	Sham and 10% solutol groups	IQ-1S can improve cerebral I/R injury after stroke by inhibiting JNK pathway
Yin et al.^131^	2016	Sprague-Dawley rats	NO/H_2_S-NBP	Oral gavage	80 and 380 mg/kg/d	7 consecutive days before MCAO	Blank and 10% solution groups	NO/H2S-NBP can improve neurobehavioral function after cerebral infarction, reduce cerebral infarction area, and improve brain edema
Sienel et al.^120^	2023	Male C57BL/6 mice	NO	Inhalation	50 ppm	Directly after reperfusion	Sham and MCAO without NO inhalation groups	Inhaled NO can reduce the occurrence of neuroinflammation
Terpolilli et al.^132^	2012	C57BL/6 mice Merino Sheep	NO	Inhalation	5-50 ppm	10 min after stroke onset 1 h after stroke onset	MCAO without any treatment	Inhaled NO selectively dilates blood vessels in the ischemic penumbra, increases collateral circulation
Guo et al.^121^	2025	Sprague-Dawley mice	GNO-OD HG@ISO-1 NPs	In situ injection	2 iL	3 d after stroke onset	Sham and PT without treatment groups	GNO-OD HG@ISO-1 NPs can reduce post-stroke motor function impairment by interfering with a variety of post-stroke injury pathways

DEA/NO: Diethanolamine NO; DETA/NONOates: (Z)-1-[N-(2-aminoethyl)-N-(2-ammonioethyl) aminio] diazen-1-ium-1,2-diolate; DMSO: dimethyl sulfoxide; GNO-OD HG@ISO-1 NPs: double-crosslinked responsive hydrogel releases NO and encapsulates ISO-1 nanoparticle; GSNO: S-nitrosogulatathione; GTN: glyceryl trinitrate; H_2_S-NBP: hydrogen sulfide-donating moieties; hMSC: human marrow stromal cell; I/R: ischemia-reperfusion; IQ-1S: ^11^H-mdeno[1,2-b]qumoxalm-11-one; JNK: c-Jun-N-terminal kinase; L-Arg: L-arginine; LA 419: a NO donor; MCAO: middle cerebral artery occlusion; NaOH: sodium hydroxide; PAMNs: a PLT membrane biomimetic nanocarrier loaded with L-arginine and γ-Fe_2_O_3_ magnetic nanoparticles; PBS: phosphate buffered solution; PEI-PO-NONOates: poly ethylenimine-based N-diazeniumdiolate NO suppliers; ppm: parts per million; ProliNO: proline NO; SIN-1: 3-morpholino-sydnonimine; SNP: sodium nitroprusside; tPA: tissue plasminogen activator; ZJM-289: a NO donor.

**Table 4:** Clinical trials of nitric oxide (NO) in ischemic stroke

Study	Year	Study type	Disease	Source of NO	Route of administration	Dosage of source of NO	Time to giving source	Control group	Results
Butterworth et al.^133^	1998	Non-RCT	AIS patients	SNP	Intravenous injection	Median dose: 0.19 μg/kg/min	Within 24 h of stroke onset	Healthy group	SNP can reduce platelet aggregation and improve regional cerebral blood flow
Rashid et al.^134^	2003	RCT	AIS patients	GTN	Transdermal	5, and 10 mg/d for 10 d	72 h within stroke onset	Standard stroke care group	Transdermal GTN lowered blood pressure, but did not improve patient outcomes
Willmot et al.^135^	2006	RCT	Patients with recent stroke	GTN	Transdermal	5 mg/d for 7 d	5 d within stroke onset	Placebo group	Transdermal GTN have no connection with changes in CBF or cerebral perfusion pressure
Woodhouse et al.^136^	2015	RCT	AIS patients	GTN	Transdermal	5 mg/d for 3 d	6 h within stroke onset	Standard stroke care group	Transdermal GTN improves functional and other clinical outcomes and reduces death and SAEs
Bath et al.^140^	2001	RCT	AIS patients	GTN	Transdermal	5 mg/d for 12 d	5 d within stroke onset	Placebo transdermal group	Transdermal administration of GTN can effectively reduce blood pressure in stroke patients
Tunnage et al.^137^	2022	RCT	Adults with ultra-acute presumed stroke	GTN	Transdermal	5 mg/d for 4 d	Immediate administration in the ambulance	Sham patch group	At 90 d, the GTN group had a better mRS score as compared to the sham group
Cheng et al.^138^	2023	RCT	AIS after thrombectomy	GTN	Intra-arterial injection	800 μg of GTN	After thrombectomy	Saline injection group	Intra-arterial administration of GTN successfully raised NOx levels
Cai et al.^139^	2024	RCT	AIS patients	GTN	Intravenous injection	5 mg in 50 mL saline for 2 d	Within 24 h of stroke onset	Saline injection group	GTN group showed superior NIHSS recovery; Non-thrombolysis patients and mild stroke patients (NIHSS < 6) had improved NIHSS scores and recovery

AIS: Acute ischemic stroke; CBF: cerebral blood flow; GTN: glyceryl trinitrate; mRS: modified Rankin scale; NIHSS: National Institutes of Health stroke scale; RCT: randomized controlled trial; SAE: serious adverse event; SNP: sodium nitroprusside.

This study assessed the risk of bias in the included animal experiments using the SYRCLE tool. In IS studies, the risk of bias for sequence generation (01) and allocation concealment (03) were predominantly “Unclear,” with only a few studies reporting explicit randomization methods. Baseline characteristics (02) showed a lower risk of bias, with most studies rated as “Yes.” However, performance and detection bias related to blinding (05, 07) and random outcome assessment (06) were significant concerns, as most studies lacked clear descriptions. Similarly, in SAH studies, sequence generation and allocation concealment were frequently “Unclear,” while blinding and outcome assessment exhibited high bias risk, except in a few studies with lower bias. Overall, the primary sources of bias in both models were randomization, blinding, and allocation concealment, whereas Incomplete outcome data (08) and selective reporting bias (09) were minimal, with most studies rated as “Yes.” (**Additional Figures 1** and **2**)

The risk of bias for all included RCTs was rigorously evaluated using the Cochrane ROB2 tool. For IS studies, three trials (42.9%) demonstrated low risk of bias across all domains. The remaining four trials (57.1%) raised some concerns. In SAH studies (*n* = 2), one trial showed low risk in all domains, while another trial exhibited some concerns due to issues in randomization, intervention deviations, outcome measurement, and selective reporting. The assessment on ICH revealed that one study was rated as having a low risk of bias across all domains. None of the RCTs were rated as “high risk” across the three evaluated groups. However, one IS clinical non-randomized controlled study was identified as having a high risk of bias upon evaluation using the ROBINS-I tool. Detailed risk-of-bias assessments are presented visually in **Additional Figures 3–6**.

### Source and Route of Nitric Oxide

**Figure 2:**
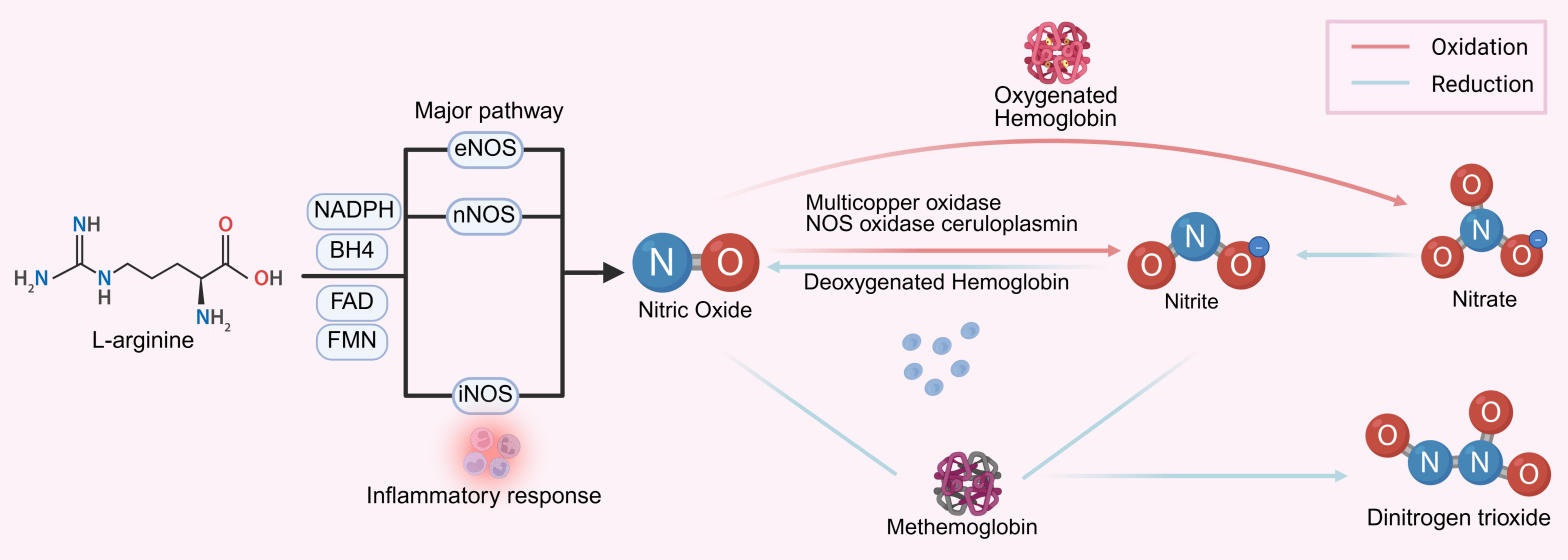
Circulation of nitric oxide (NO). There are two primary endogenous pathways for NO production: the NO_3_^−^-NO_2_^−^-NO pathway and L-Arg/NOS pathway. In the L-Arg/NOS pathway, L-Arg, with the assistance of cofactors, such as NADPH, BH4, FMN, and FAD, binds to eNOS or nNOS to produce NO. Under inflammatory conditions, the expression of iNOS is upregulated, resulting in the generation of large amounts of NO through iNOS. The NO produced is oxidized by oxyhemoglobin into nitrate. Under specific conditions, nitrate is converted into nitrite once it enters the bloodstream. In oxygen-rich environments, NO is catalyzed into nitrite by multicopper oxidases and NOS oxidase ceruloplasmin. In hypoxic conditions, nitrite can interact with methemoglobin to form the more stable N_2_O_3_, which is capable of diffusing through red blood cell membranes and can be stored in the vasculature for extended periods. Created with BioRender.com. BH4: Tetrahydrobiopterin; eNOS: endothelial nitric oxide synthase; FAD: flavin adenine dinucleotide; FMN: flavin mononucleotide; iNOS: inducible nitric oxide synthase; L-Arg: L-arginine; N_2_O_3_: dinitrogen trioxide; NADPH: nicotinamide adenine dinucleotide phosphate; NO_2_^−^: nitrite ion; NO_3_^−^: nitrate ion; nNOS: neuronal NOS; NOS: nitric oxide synthase.

The production of endogenous NO primarily occurs through two pathways: the NOS-dependent pathway and the inorganic salt pathway.^30^ **Figure 2** shows the circulation of NO in the vascular.

### Nitrate–nitrite–nitric oxide pathway

NO is a potentially toxic gaseous molecule that cannot exist for prolonged periods within biological systems. Once generated, NO rapidly binds to oxygenated Hb, forming relatively less toxic nitrogen oxide derivatives.^31^,^32^ Under specific conditions, these derivatives can react to regenerate NO, thus serving as an important storage reservoir for NO in the body.^31^,^32^ Additionally, certain derivatives possess their own reactivity and act as signaling molecules involved in various biological processes; these active NO derivatives are referred to as reactive nitrogen species.^33^ This phenomenon lays the groundwork for scientists to develop NO carriers or NO derivatives as therapeutic agents.^34^

The most common reactive nitrogen species in the body are primarily nitrates and nitrites, whose sources largely depend on dietary intake. Certain vegetables, such as spinach, lettuce, and beets, are rich in nitrates, while nitrites mainly originate from cured foods.^35^ Ingested nitrates are primarily excreted via urine, with the kidneys, intestines, and salivary glands being the main sites for nitrate reabsorption.^36^ Notably, the reabsorption in the oral salivary glands is significant, with over a quarter of exogenous nitrates being reduced to nitrites by the symbiotic facultative anaerobic bacteria in the oral cavity.^37^ Subsequently, nitrites combine with hydrogen ions in the acidic environment of the stomach to form NO. The entero-salivary circulation allows plasma nitrate levels to be maintained at elevated levels for extended periods. This pathway, known as the nitrate–nitrite–NO pathway, complements the classical L-Arg/NOS pathway in the body, facilitating NO production and enhancing its bioavailability.^31^

A study has indicated that exogenous supplementation of nitrates can significantly reduce baseline blood pressure levels in elderly individuals with prehypertension.^36^ Moreover, no significant tolerance has been observed with exogenous nitrate supplementation compared to nitroglycerin.^38^ Furthermore, recent research highlights that exogenous nitrite supplementation can enhance NO bioavailability, neutralize excess superoxide produced during oxidative stress, and improve the bioactivity of tetrahydrobiopterin (BH4), thereby improving endothelial function in patients with chronic obstructive pulmonary disease.39 These findings provide a physiological basis for the use of nitrates and nitrites as sources of exogenous NO.

### L-arginine/nitric oxide synthase pathway

Endogenous NO is primarily generated from L-Arg under the catalytic action of NOS. There are three distinct types of NOS in the human body: endothelial NOS (eNOS), neuronal NOS (nNOS), and inducible NOS (iNOS).^40^ The structure of NOS includes a reductase domain at the C-terminus and an oxygenase domain at the N-terminus. The N-terminal oxygenase domain possesses enzymatic activity, allowing it to bind heme, BH4, and L-Arg. The C-terminal reductase domain contains binding sites for flavin adenine dinucleotide, flavin mononucleotide, and nicotinamide adenine dinucleotide phosphate, and is linked to the N-terminal domain via a calmodulin (CaM) recognition site, forming a complete structure.^41^ All three types of NOS utilize L-Arg as a substrate and work in conjunction with cofactors (BH4, flavin adenine dinucleotide, flavin mononucleotide, and nicotinamide adenine dinucleotide phosphate) to synthesize endogenous NO, with the synergistic actions of CaM and Hb. Notably, the binding of eNOS and nNOS to CaM is calcium-dependent, whereas iNOS can bind to CaM even at very low calcium concentrations, which is one of the main differences between them.^42^

There are certain differences in the expression of the three NOS types in vivo. Studies have shown that eNOS and nNOS are often co-expressed, mediating the release of low doses of NO to exert protective effects such as neuronal signaling, vascular endothelial relaxation, and inhibition of platelet aggregation.^43^,^44^,^45^ In contrast, iNOS is primarily expressed in response to inflammatory stimuli, generating high doses of NO in a short period and participating in a range of inflammatory stress responses, mediating the cytotoxic effects of NO.^45^ Under physiological conditions, eNOS is primarily expressed in vascular endothelial cells, participating in the physiological process of endothelial relaxation. Additionally, some studies have indicated that certain circulating blood cells carry the transcription products or proteins of eNOS.^46^,^47^ nNOS is mainly expressed in normal neuronal cells, with evidence suggesting that a small amount of nNOS expression also exists in astrocytes.^48^ Interestingly, scientists have found that when astrocytes and neurons combine, a small amount of eNOS expression can be detected.^49^,^50^,^51^ In immune cells such as neutrophils and macrophages, which mediate extensive inflammatory immune responses, the expression of iNOS can be monitored.^52^

Under physiological conditions, endothelial cells and circulating blood cells express eNOS, while neurons and skeletal muscle cells express nNOS in a constitutive manner, locally producing small amounts of NO that participate in processes such as the dilation of small blood vessels and the transmission of neuronal signals.^53^ In contrast, iNOS plays a role in the non-specific immune response against pathogens by locally generating NO to exert strong oxidative effects, damaging pathogen structures and aiding immune cells in pathogen elimination.^52^ Due to its strong oxidative properties, NO typically does not persist in the body for long. Oxygenated Hb rapidly clears local NO to prevent accumulation that could lead to tissue damage. Thus, the endogenous production of NO mediated by the three NOS types primarily serves protective cellular functions under physiological conditions.^47^

In pathological conditions, such as ischemic injury, the three NOS alter their normal physiological characteristics, resulting in the local production and accumulation of large amounts of NO over a short period, which subsequently damages NO-related functions and affects downstream responses, causing further tissue injury.^54^ eNOS and nNOS are calcium-dependent NOS. Under hypoxic conditions, changes in the cellular microenvironment and the secretion of numerous inflammatory factors can impair the normal function of eNOS and nNOS, inhibiting their activity and significantly reducing local NO production. The secretion of iNOS is typically inducible, often initiated by immune defense cells such as neutrophils in the presence of pathogens to participate in the eradication of bacteria and other microorganisms. However, when local tissue is in a hypoxic state, initial injury triggers an inflammatory response, leading to an increase in the expression of inflammatory factors, particularly pro-inflammatory cytokines such as tumor necrosis factor-alpha and interleukins, which can enhance iNOS activity and result in the rapid production of large amounts of NO. Additionally, due to the hypoxic state, nitrites in the blood may convert to NO, further contributing to NO accumulation and tissue damage. This appears to complement the substantial NO produced by iNOS. However, due to differing temporal sequences, this process may lead to tissue damage at different stages.^55^

Meanwhile, NOS plays a critical role in various disease states. For example, knockout of the NOS3 gene in mice leads to primary hypertension, and the proliferation and apoptosis of some tumor cells are also linked to NO.^56^ Furthermore, nNOS is involved in neuronal apoptosis, morphological changes, and the pathogenesis of certain neurodegenerative diseases, such as Alzheimer’s and Parkinson’s diseases.^57^ Therefore, understanding the role of NO in the three major types of strokes necessitates a comprehensive understanding of the functions of these three NOS types.^51^

### Role of Nitric Oxide and Its Donors in Subarachnoid Hemorrhage

**Figure 3:**
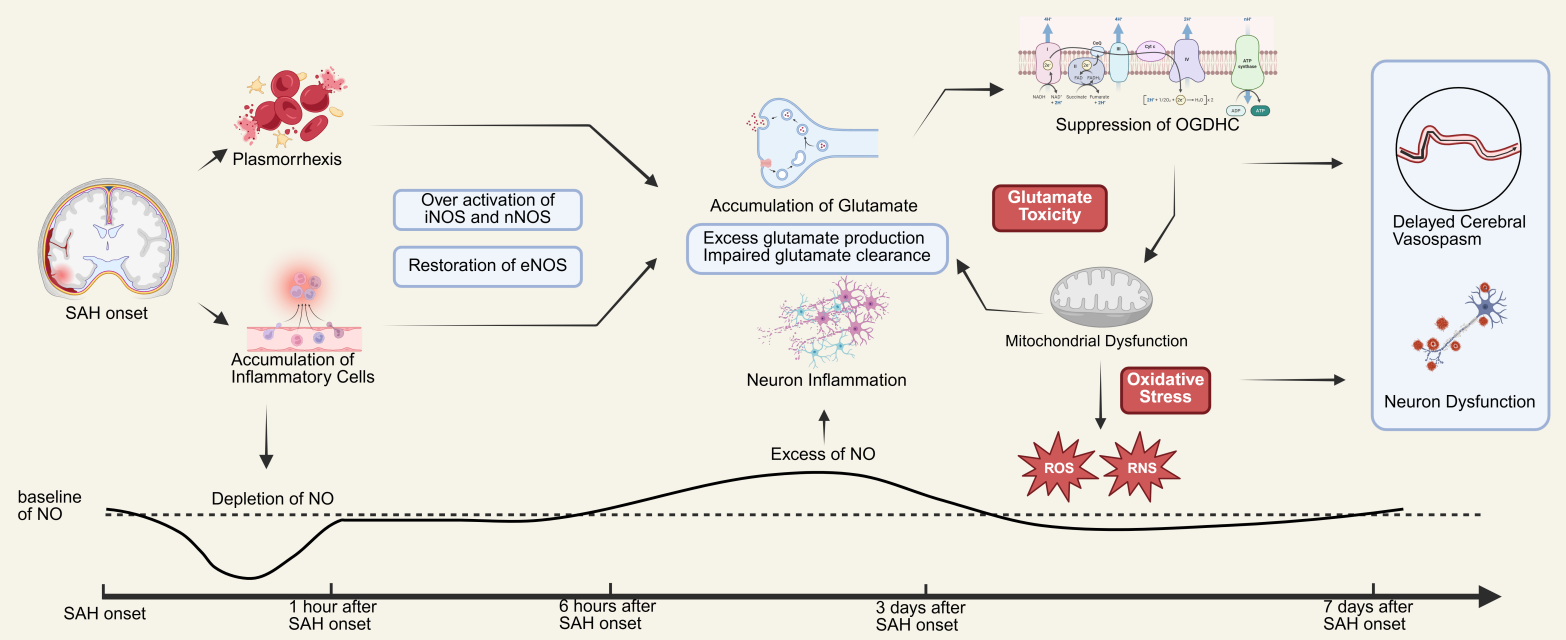
Role of nitric oxide (NO) in subarachnoid hemorrhage (SAH). SAH is triggered by the rupture of intracranial blood vessels, followed by the breakdown of red blood cells, the accumulation of inflammatory cells, and the onset of neuroinflammation, leading to rapid depletion of local NO. This process primarily occurs within the first hour after SAH onset. Subsequently, the expression of eNOS and iNOS is activated, gradually restoring local NO levels to baseline. However, after 6 hours, the sustained overexpression of NO induces toxic effects, exacerbating glutamate toxicity and impairing the mitochondrial electron transport chain, resulting in mitochondrial dysfunction and further aggravating glutamate toxicity. In addition, the excessive production of reactive oxygen and nitrogen species triggers localized oxidative stress, further reducing NO bioavailability, ultimately leading to vasospasm and neuronal injury. This process persists until the 7^th^ day post-SAH, after which NO levels return to baseline, and normal NO utilization gradually resumes. This restoration of NO also provides the physiological basis for the therapeutic effects of exogenous NO supplementation in alleviating vasospasm. Created with BioRender.com. eNOS: Endothelial nitric oxide synthase; iNOS: inducible nitric oxide synthase; NOS: nitric oxide synthase; OGDHC: 2-oxoglutarate dehydrogenase complex; RNS: reactive nitrogen species; ROS: reactive oxygen species.

Existing research indicates that endogenous NO exhibits systematic variations within 7 days following SAH.^58^ Within 10 minutes after the onset of SAH, the levels of NO and its metabolites in the brain significantly decrease, contrasting with the increase in NO levels observed after IS. This decline may be attributed to factors such as the clearance by Hb and the binding of free radicals. Concurrently, the stimulation of blood to brain tissue following SAH triggers neuroinflammation, inhibiting the expression of eNOS while having a lesser impact on the expression of nNOS and iNOS.^59^ During the initial stages of SAH (from 10 minutes to several hours), the rapid depletion of NO impairs the NO-dependent vasodilatory function. However, arterial responsiveness to NO remains intact. Therefore, the administration of exogenous NO donors during this period can significantly alleviate CVS. In the subsequent 1 to 6 hours, the expression of nNOS and iNOS gradually increases, and eNOS expression returns to normal levels, leading to a rebound in NO levels to baseline. However, despite the persistent vasospasm, the application of exogenous NO donors may not effectively relieve vascular constriction and could instead provoke neurotoxic effects due to NO overload, making this phase an unsuitable time for pharmacological intervention. From 7 to 72 hours, NO levels significantly exceed baseline values, primarily due to the increased expression of iNOS. This excess NO exerts detrimental effects on the brain by directly inhibiting the function of the mitochondrial electron transport chain in neural cells. Additionally, the glutamate toxicity triggered by SAH synergizes with NO to further impair mitochondrial function, leading to the accumulation of glutamate and exacerbating neurotoxicity.^60^,^61^ Furthermore, as a free radical, NO can induce oxidative stress, resulting in DNA damage and exacerbating neuronal apoptosis by altering ion channels. Thus, the use of NO donors during this stage may worsen damage to neural tissue. Between days 3 and 7 post-SAH, as various physiological functions gradually restore, NO levels gradually return to baseline. However, delayed CVS often occurs during this period. At this point, the application of exogenous NO donors may have a beneficial effect on delayed CVS.^62^,^63^,^64^,^65^,^66^ The role of NO in SAH is shown in **Figure 3**.

Early research suggested that CVS is a major cause of severe complications in patients with SAH, leading most studies to focus on the role of NO in delayed CVS following SAH. Vasospasm, a condition marked by impaired vascular dilation and constriction, naturally prompts consideration of NO, a pivotal molecule in the regulation of vascular tone. The involvement of NO in SAH was initially elucidated by Kanamaru et al.^67^ through a canine model. They demonstrated that the extravasation of Hb disrupts endothelial function, thereby inducing vasospasm following SAH. This seminal work not only underscored the critical role of NO in the pathophysiology of SAH but also laid the groundwork for further investigation into the role of NO in this context. Subsequent studies have further established NO as an essential regulator of endothelial function.^44^,^68^

In 1997, Pluta et al.^69^ found that acute internal carotid artery injection of non-adenosine-non-oxygenated NO donors (NONOates) could reverse CVS in a primate model of SAH. In 1998, Wolf et al.^70^ discovered that administering long-acting NO donors seven days after SAH in dogs could improve the occurrence of vasospasm. Kiriş et al.^71^ in 1999 demonstrated in a rabbit model that intravenous injection of S-nitroso-N-acetyl penicillamine alleviated delayed CVS following SAH. By 2000, Pluta et al.^72^ reported that supplementing the NO precursor L-Arg improved local cerebral blood flow after SAH, a finding further supported by Sun et al.^73^ in 2003. Similarly, Göksel et al.^74^ reached comparable conclusions in a rabbit study in 2001. As research progressed, more NO donors and various administration methods were introduced. For instance, Sönmez et al.^75^ in 2002 applied a spermine/NO complex in a rabbit SAH model, finding that internal carotid artery injection effectively alleviated post-SAH vasospasm. Gabikian et al.^76^ utilized a new NO carrier, 20% diethyl triamine-NONOate/ethyl vinyl acetate, which improved local NO concentration and reduced vasospasm in a mouse model by direct implantation into the lesion area. In 2004, Pradilla et al.^77^ reported that low-dose diethylenetriamine-NO exerted no significant effect on cerebrovascular dilation, whereas high-dose administration produced a marked effect, indicating a dose-dependent response. Sodium nitroprusside (SNP), another organic NO donor, was shown by Lambert et al.^78^ in 2001 to reverse sympathetic nerve excitation following SAH and improve vasospasm. However, Macdonald et al.^79^ in 2002 reported high-dose SNP-induced smooth muscle relaxation via intracisternal infusion but did not significantly improve CVS, possibly due to its toxicity. In 2016, Lilla et al.^80^ found that low-dose intravenous SNP improved early perfusion deficits, reduced neuronal injury, and lowered blood pressure in a rabbit model of SAH. These findings suggest that NO donors exhibit a dual dose-dependent effect in managing post-SAH vasospasm, with efficacy at low doses but potential toxicity at higher doses. Related animal research was summarized in **Table 1**.^69^,^70^,^71^,^72^,^73^,^74^,^75^,^76^,^77^,^78^,^79^,^80^,^81^,^82^,^83^,^84^,^85^,^86^,^87^,^88^,^89^,^90^,^91^,^92^,^93^

In recent years, research on SAH has shifted its focus from CVS to neuroinflammation, microcirculatory disturbances, BBB damage, and dysregulation of cerebral vascular autoregulation. In 2013, Li et al.^83^ found that continuous inhalation of 60 ppm NO for 8 hours post-SAH in mice could reduce neuroinflammation and improve recovery following SAH. In 2016, Terpolilli et al.^84^ observed improvements in microvascular vasospasm in SAH mice through inhalation of 50 ppm NO, which exhibited neuroprotective properties and facilitated neurological rehabilitation.

Clinical trials validated these theories. In 2004, Reinert et al.^94^ found that transdermal nitroglycerin positively influences cerebral blood flow. A RCT further demonstrated that intravenous sodium nitrite infusion enhances NO bioavailability and effectively improves cerebral hypoperfusion in patients with SAH.^95^ Currently, a large clinical trial related to the function of transdermal GTN in stroke patients, named Efficacy of Nitric Oxide in Stroke (ENOS) clinical trial, is still ongoing. Related clinical trials in the role of NO in SAH was summarized in **Table 2**.^94^,^95^

### Role of Nitric Oxide and Its Donors in Intracerebral Hemorrhage

ICH is characterized by bleeding within the brain parenchyma due to the rupture of small blood vessels. Since SAH is a specific type of cerebral hemorrhage, it has been discussed separately in the preceding sections. The role of NO in cerebral hemorrhage remains relatively complicated. In 2016, Guo et al.^2^ reviewed the temporal dynamics of NO following traumatic brain injury. They proposed that eNOS and nNOS were significantly activated within 5–30 minutes post-injury, leading to a substantial increase in NO production. Subsequently, due to insufficient eNOS activation, NO levels markedly declined and remained low until approximately 6 hours post-injury. Thereafter, NO levels gradually increased, likely due to endogenous regulatory mechanisms within the brain, and reached a peak between 20 and 24 hours after injury. This later phase was closely associated with iNOS.

Previous studies have primarily focused on the relationship between NO levels and the prognosis of ICH. Kim et al.96 demonstrated that eNOS-deficient mice subjected to ICH exhibited reduced brain edema. In a subsequent review, Li et al.^97^ summarized that following ICH, Hb-induced stimulation activates perihematomal glial cells and infiltrating neutrophils, resulting in the overexpression of iNOS and nNOS. This leads to excessive NO production, which exerts a secondary detrimental effect on vulnerable brain tissue, exacerbating neural injury. The underlying mechanism is closely linked to the asymmetric dimethylarginine/dimethylarginine dimethylaminohydrolase pathway, with nuclear factor kappa B also playing a role in the associated inflammatory processes.^98^ Furthermore, Chiang et al.^99^ reported that elevated NO levels in cerebrospinal fluid and lower Glasgow Coma Scale scores upon admission were indicative of more pronounced NOS activation. In contrast, Rashid et al.^100^ suggested that low systemic NO levels prior to ICH onset may serve as a predictive marker for pre-existing arterial damage. The primary etiology of cerebral hemorrhage is often associated with hypertension, and NO plays a significant role in the pathophysiology of hypertension, which suggests that NO supplementation could be a potential intervention to reduce the risk of hypertensive cerebral hemorrhage. Nevertheless, this topic is not the focus of this article, and thus it will not be reviewed in detail.

Research related to exogenous NO supplementation was only mentioned in the 2015 ENOS clinical trial subgroup analysis. Krishnan et al.^101^ found that in patients with cerebral hemorrhage, transdermal administration of 5 mg of GTN daily for 7 days within 6 hours of onset effectively and safely reduced blood pressure; however, no significant improvements in neurological recovery or other prognostic outcomes were observed. It indicates that the transdermal administration of GTN within 6 hours may not be sufficient to effectively restore the deficiency of NO. However, the available evidence is currently inadequate to support the formulation of further clinical pharmacological guidelines. Therefore, further clinical studies are needed to explore the relationship between the administration of NO and its donors and the treatment of ICH, in order to clarify the potential value of supplementing NO or its donors after the onset of ICH.

### Role of Nitric Oxide and Its Donors in Ischemic Stroke

IS is the most common type of stroke. For many years, researchers have focused on strategies to salvage the ischemic penumbra, thereby reducing neuronal damage. Currently, intravenous administration of thrombolytic agents, such as recombinant tissue plasminogen activator, is the primary method for treating IS, and endovascular mechanical thrombectomy is also commonly employed for reperfusion.^16^ However, while reperfusion can rescue some ischemic brain tissue, it is also associated with the risk of reperfusion injury. NO, an important vascular regulatory factor, has long been recognized for its role in ischemia-reperfusion. Numerous therapeutic strategies aimed at targeting the NOS pathway have been developed. In 2012, Terpolilli et al.^102^ conducted an extensive review that encapsulated the pertinent research findings. Consequently, the present review is dedicated solely to examining studies concerning the direct administration of NO.

**Figure 4:**
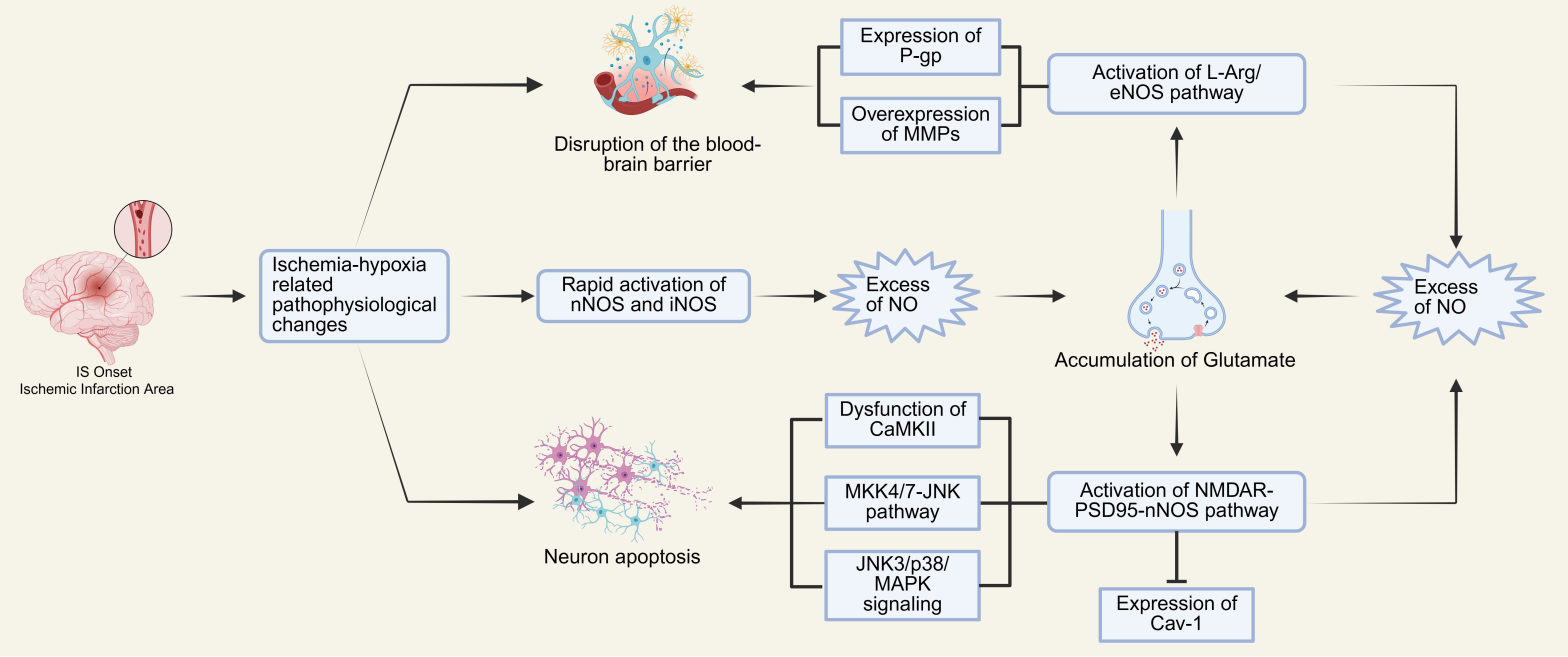
Role of nitric oxide (NO) in ischemic stroke (IS). After the onset of IS, a series of pathophysiological alterations associated with ischemia and hypoxia occurred in the infarct core. These changes led to the rapid activation of nNOS and iNOS, resulting in excessive NO production. The excess NO facilitated the accumulation of glutamate at synaptic clefts, thereby inducing glutamate excitotoxicity. Subsequently, the NMDAR-PSD95-nNOS signaling pathway was activated by the accumulation of glutamate, which not only inhibited the normal activity of CaMKII but also activated the JNK3/p28/MAPK and MKK4/7-JNK signaling pathways. Furthermore, this process suppressed the expression of Caveolin-1, exacerbating neuronal death. Additionally, the L-Arg/eNOS pathway was activated as well, leading to the overexpression of P-gp and MMPs. These events collectively contributed to the aggravation of blood-brain barrier damage induced by ischemia. Created with BioRender.com. CaMKII: Ca^2+^/calmodulin dependent protein kinase IIα; cav-1: caveolin-1; eNOS: endothelial nitric oxide synthase; JNK3: c-Jun-N-terminal kinase 3; L-Arg: L-arginine; MAPK: mitogen-activated protein kinase; MKK4/7: mitogen-activated protein kinase kinase 4/7; MKL3: mixed linage kinase 3; MMP: matrix metalloproteinase; NMDAR: N‐methyl‐D‐aspartate receptor; nNOS: neuronal nitric oxide synthase; P-gp: P-glyco protein; PSD95: postsynaptic density protein 95.

Interestingly, NO exerts its vasodilatory effects primarily in the ischemic penumbra during IS, suggesting that the application of exogenous NO or NO donors could effectively alleviate microvascular spasms in this area, thereby improving patients’ long-term outcomes.^19^ However, in the core infarct region, NO acts as a detrimental factor, directly involved in the pathophysiological changes occurring in that area.^103^ Following infarction, nNOS is rapidly activated, and as ischemia-induced neuroinflammation escalates, iNOS is also activated. Studies indicate that nNOS generates large amounts of NO after cerebral ischemic injury, inducing the aggregation of inflammatory cells and promoting substantial expression of iNOS, which further increases local NO accumulation via the iNOS pathway.^104^,^105^ The rapid production of excessive NO stimulates the accumulation of glutamate, leading to glutamate toxicity and exacerbating brain tissue damage. Additionally, acute ATP depletion caused by ischemia-reperfusion alters the transmembrane potential, and in conjunction with glutamate toxicity, promotes Ca²^+^ influx and the formation of reactive nitrogen and oxygen species. These factors, along with excess NO, contribute to neuronal death. NO produced by nNOS activates the N‐methyl‐D‐aspartate receptor (NMDAR)-postsynaptic density protein-95 (PSD95)-nNOS pathway, triggering delayed neuronal injury.^106^ Excessive NO can also inhibit the autophosphorylation of Ca²^+^/calmodulin-dependent protein kinase II (CaMKII) through S-nitrosylation, leading to CaMKII dysfunction and further aggravating brain tissue injury. While some studies suggest that Ca²^+^ can activate CaMKII, which in turn phosphorylates nNOS and inhibits NO production, current research generally indicates that the inhibitory effect of nNOS on CaMKII takes precedence over the opposite. Therefore, the NO generated by nNOS primarily exhibits damaging effects on neurons. Furthermore, excess NO may indirectly activate the mitogen-activated protein kinase kinase 4/7-c-Jun-N-terminal kinase 3 signaling pathway by activating mixed linage kinase 3 and apoptosis signal-regulatory kinase 1, ultimately leading to neuronal apoptosis.^107^ Glutamate toxicity can also activate the L-Arg/eNOS pathway, resulting in excessive NO and peroxynitrate production, which induces overexpression of P-glycoprotein and matrix metalloproteinases, thereby exacerbating BBB damage.^20^,^24^ Related mechanisms are illustrated in **Figure 4**.

SNP was the pioneering pharmacological agent employed to enhance cerebral blood flow post-IS.^108^ Beyond SNP, other NO-releasing compounds, such as SIN-1,^108^,^109^ isosorbide dinitrate,^110^ proline NO,^111^ S-nitroso glutathione^112^ and LA 419,^113^,^114^ have demonstrated potential in augmenting cerebral blood flow within the ischemic penumbra following an IS, thereby potentially improving stroke outcomes. Besides, Greco et al.^115^ found that systemic administration of GTN prior to middle cerebral artery occlusion (MCAO) in mouse models reduced the area of cerebral infarction. However, the protective effects were limited due to the potential for tolerance development with GTN. A recent animal experiment demonstrated that a specific dosage of GTN, when used in combination with thrombolytic agents after ischemia–reperfusion, significantly improved the infarct area, although high doses increase the risk of bleeding.^116^ Sorby-Adams et al.^117^ further validated that transdermal delivery method showed limited effectiveness. Additionally, the propensity for GTN to induce tolerance in vascular dilation limits its efficacy, prompting researchers to explore other NO donors. For instance, in 2020, Li et al.^118^ developed a polyethyleneimine-based magnetic nanoparticle carrier loaded with L-Arg and γ-Fe_2_O_3_, which could be administered via the tail vein in mice. These particles quickly localized to the ischemic area, increasing local NO levels, promoting vasodilation, reducing platelet aggregation, and delaying thrombosis. Zhang et al.^119^ introduced a novel NO donor, polyethyleneimine-based n-diazole diol NO donors, capable of directly releasing NO at the ischemic area. They found that administering NONOates to MCAO mice significantly reduced the area of ischemic brain tissue. Furthermore, Sienel et al.^120^ discovered that inhalation of NO directly administered to MCAO mice could significantly alleviate the neuroinflammatory response in brain tissue after ischemia–reperfusion by modulating leukocyte adhesion to endothelial cells. In the latest published literature, Guo et al.^121^ developed a dual-crosslinked responsive nanoparticle platform capable of NO. By delivering NO *in situ*, this platform effectively scavenges free radicals in the ischemic penumbra, alleviates neuroinflammation, and promotes stroke recovery. This also signifies that novel nanomaterials are increasingly playing a more significant role in the delivery of NO. Animal research related to the role of NO in IS has been summarized in **Table 3**.^108^,^109^,^110^,^111^,^112^,^113^,^114^,^115^,^116^,^117^,^118^,^119^,^120^,^121^,^122^,^123^,^124^,^125^,^126^,^127^,^128^,^129^,^130^,^131^,^132^

Clinical trials on the role of NO and its donors in IS also began 30 years ago. In 1998, the first clinical trial of NO donors in stroke was conducted by Butterworth et al.^133^ This study involved the continuous administration of SNP to patients with acute stroke, and a reduction in infarct size was observed at an optimal dosage. However, this dosage was also closely associated with a decrease in blood pressure. GTN is currently the most widely used organic NO donor in clinical practice, with significant findings from related clinical studies. Clinical trials related to GTN were initiated as early as 2003, yet no positive results have been reported. Rashid et al.^134^ suggested that such results may be attributable to the small sample size. However, their study validated the safety of GTN, also serving as one of the supports for the large-scale ENOS clinical trial. In 2006, Willmot et al.^135^ conducted a 7-day treatment with transdermal GTN at a dosage of 5 mg daily in patients with IS within 5 days of onset, finding no significant association between transdermal GTN and changes in cerebral blood flow or cerebral perfusion pressure. In 2015, Woodhouse et al.^136^ in the ENOS study found that administering transdermal GTN at a daily dose of 5 mg within 6 hours after the onset of IS for 3 days resulted in improved neurological function and clinical outcomes, significantly reducing mortality. Tunnage et al.^137^ observed in large clinical trials that administering transdermal GTN to patients with suspected IS led to higher modified Rankin scale scores at 90 days, indicating better long-term prognosis. In 2023, Cheng et al.^138^ reported that arterial injection of 800 μg GTN in IS patients undergoing thrombectomy effectively increased local NO levels in the brain within a short time and served as a safe measure to prevent vascular spasm following endovascular intervention. In 2024, Cai et al.^139^ found that dissolving 5 mg GTN in 50 mL of saline and continuously infusing it intravenously at a rate of 0.4 mg/h for 12.5 hours, followed by two days of treatment, significantly improved National Institutes of Health stroke scale scores, particularly benefiting non-thrombolysis patients or those with mild strokes. This suggests that GTN may become one of the effective drugs for improving prognosis in IS patients. In contrast, research on inorganic NO donors is relatively scarce. The results of clinical trials in the role of NO in IS are listed in **Table 4**.^133^,^134^,^135^,^136^,^137^,^138^,^139^,^140^

### Mechanistic Insights into Nitric Oxide in Stroke

#### Neuroprotection and neurotoxicity

NO exerts essential neurotransmitter and neuroprotective roles at physiological levels, whereas excessive NO production induces significant neurotoxicity. NO participates in protein kinase B and cyclic adenosine monophosphate responsive element-binding protein-mediated neuroprotection via the cyclic guanosine monophosphate and protein kinase G pathways. Additionally, NO reduces neuronal excitatory stimuli by S-nitrosylating NMDAR subunits, thereby preventing excessive activation of nNOS. Furthermore, NO upregulates hemoxygenase expression, promoting the formation of antioxidant and antinitrosative molecules such as bilirubin and biliverdin, thereby indirectly exerting cytoprotective effects.^141^ As summarized earlier, in patients with SAH, NO and its related donors can alleviate delayed CVS by dilating cerebral vessels and improving cerebral blood flow, thereby conferring neuroprotection. In IS patients, particularly those undergoing vascular recanalization, NO enhances cerebral perfusion, salvaging more ischemic penumbra. Beyond its vascular actions, eNOS-derived NO exhibits anti-thrombotic properties by inhibiting platelet aggregation and adhesion to the vascular endothelium.

Most of the neurotoxic effects of NO are mediated by peroxynitrite, manifesting as dysregulated glutamatergic neurotransmission.^142^ Excessive NO leads to synaptic glutamate accumulation post-stroke, disrupting NMDAR signaling and subsequently increasing vascular permeability and BBB dysfunction.

Excessive NO production from nNOS and inducible iNOS contributes significantly to stroke brain damage. Neurotoxic effects of NO are mediated by peroxynitrite, a highly reactive nitrogen species. This potent oxidant induces protein nitration, lipid peroxidation, and DNA damage, while simultaneously impairing mitochondrial function through inhibition of key respiratory chain enzymes. The resulting energy failure exacerbates neuronal death and expands the ischemic core.^142^ iNOS-derived NO, produced by activated microglia, astrocytes, and infiltrating leukocytes 12–24 hours post-stroke, sustains neuroinflammation and promotes BBB disruption through multiple mechanisms, including tight junction protein degradation and matrix metalloproteinase activation. This BBB breakdown facilitates the entry of harmful blood components into the brain parenchyma, worsening vasogenic edema and secondary neuronal injury.^143^ These changes ultimately exacerbate infarct expansion in IS and perilesional edema in hemorrhagic stroke, as previously summarized.

#### Neurotransmission and neurosynaptic plasticity

NO exerts a paradoxical influence on stroke pathophysiology through its interaction with NMDAR-dependent synaptic plasticity. Under physiological conditions, NO produced by neuronal nNOS acts as a retrograde messenger, facilitating presynaptic glutamate release and supporting NMDAR-dependent long-term potentiation, which is essential for learning and memory in the hippocampus and neocortex.^144^,^145^

During ischemia, however, excessive glutamate release and NMDAR overactivation trigger pathological calcium influx and a surge in NO production. At these concentrations, NO shifts from a physiological modulator to a neurotoxin, reacting with superoxide to generate peroxynitrite, which induces protein nitration, lipid peroxidation, mitochondrial dysfunction, and DNA damage, thereby amplifying excitotoxicity.^143^,^146^ Excess NO also disrupts synaptic integrity by modifying NMDARs and related proteins, impairing long-term potentiation and contributing to post-stroke cognitive decline.^141^,^147^ Furthermore, delayed induction of iNOS in glial and immune cells aggravates inflammation, BBB breakdown, and cerebral edema.^148^ This duality highlights a major therapeutic challenge: global inhibition of NO is detrimental, as eNOS-derived NO maintains cerebral blood flow and exerts neuroprotection. Cognitive decline following stroke significantly affects patient prognosis and quality of life.^149^ Therefore, therapeutic strategies targeting the enhancement of NOS activity or direct administration of NO may offer promising interventions for post-stroke cognitive impairment. Clarifying the spatiotemporal and concentration-dependent effects of NO on NMDAR function remains critical for the development of neuroprotective strategies aimed at preserving cognitive outcomes after stroke.

#### Cellular plasticity

NO is a critical modulator of post-stroke cellular plasticity, with effects highly dependent on its source and concentration. Low levels of eNOS-derived NO promote neurogenesis in the subventricular zone and hippocampal dentate gyrus by enhancing neural stem/progenitor cell proliferation and survival via cyclic guanosine monophosphate-dependent pathways and VEGF induction. In contrast, high levels of iNOS-derived NO inhibit proliferation and differentiation, limiting endogenous repair.^150^,^151^ Structural reorganization, including axonal sprouting and dendritic remodeling, is also regulated by NO. Through cyclic guanosine monophosphate/protein kinase G signaling, NO modulates growth cone dynamics and cytoskeletal remodeling, facilitating neuronal rewiring during recovery. However, excessive NO and chronic inflammation create an inhibitory environment that impairs neurite outgrowth.^152^ eNOS-derived NO further supports angiogenesis by stimulating endothelial proliferation, migration, and tube formation via VEGF pathways, linking vascular repair with neurogenesis and synaptic plasticity. Conversely, nNOS- and iNOS-derived NO can compromise endothelial integrity, reducing these benefits.^146^ Glial responses are similarly influenced by NO. Moderate astrocyte and microglial activation contribute to tissue repair, while sustained iNOS-derived NO promotes neuroinflammation, microglial activation, and release of pro-inflammatory cytokines, hindering plasticity. NO also modulates astrocytic glutamate uptake and trophic factor release, shaping neural network remodeling.^146^,^153^

#### Neuroinflammation

Recent investigations have increasingly concentrated on the phenomenon of neuroinflammation subsequent to stroke.^154^,^155^ Neuroinflammation represents an unavoidable pathological alteration during the stroke recovery process, and when left unchecked, it can result in suboptimal neurological outcomes.^156^ Prior research has established that inflammatory stimuli lead to a marked upregulation of various NOS isoforms within cells, culminating in the production of substantial quantities of NO, which may exacerbate tissue damage.^157^ However, a study conducted by Seinel et al.^120^ demonstrated that administering 50 ppm of inhaled NO to mice post-stroke resulted in elevated nitration levels in both peripheral blood and brain tissue, while significantly attenuating the release of inflammatory mediators such as interleukin-6 and tumor necrosis factor-alpha. Furthermore, in mice subjected to MCAO and treated with inhaled NO, a reduction in eNOS expression was observed, whereas nNOS expression remained unchanged.^120^ This suggests that the heightened secretion of inflammatory factors following stroke may drive the upregulation of eNOS in adjacent endothelial cells.^158^,^159^ In contrast, various studies have demonstrated that under normal physiological conditions, NO synthesized by eNOS is adequate to prevent leukocyte adhesion. Nonetheless, when this delicate equilibrium is disrupted and excessive NO is produced, its inhibitory effects are diminished, resulting in increased vascular permeability.^160^,^161^ This underscores the critical balance of NO levels within the body. Disruption of this balance can convert NO’s protective effects into detrimental actions, thereby exacerbating inflammatory processes and causing tissue damage. Historically, iNOS has been regarded as a form of NOS that is activated by inflammation and produces NO over prolonged periods, thus representing an inflammatory phenotype.^162^,^163^ However, recent evidence suggests that the absence of the iNOS gene may worsen symptoms in Alzheimer’s disease mouse models.^164^ Furthermore, inhibition of iNOS has been shown to ameliorate clinical symptoms in Parkinson’s disease models by preserving dopaminergic neurons.^165^ Furthermore, certain researchers have identified that iNOS can effectively reduce neurotoxicity within an *in vitro* co-culture system comprising neurons and glial cells.^166^

Collectively, these studies highlight the intricate role of NO in neuroinflammation, neuroprotection, neurotoxin, neurotransmission and cellular plasticity. The effects of NO are not merely binary; instead, the precise biological outcomes associated with different concentrations of NO require further elucidation.

### Clinical Research Registration of Nitric Oxide and Its Donors in Stroke

We systematically searched ClinicalTrials.gov and the Chinese Clinical Trial Registry up to August 30, 2025 using the terms “nitric oxide” OR “nitric oxide donor” OR “glyceryl trinitrate” OR “sodium nitroprusside” OR “isosorbide mononitrate” AND “stroke.” Following a manual review, a total of 14 clinical trials related to the application of NO and its donors in stroke treatment were identified on ClinicalTrials.com. A summary of these trials is provided in **Additional Table 1**. Additionally, no registered clinical trials meeting the inclusion criteria were found in the Chinese Clinical Trial Registry.

**Additional Table 1:** Registered clinical research related to nitric oxide and its donors in stroke

NCT No.	Study title	Study type	Interventions	Study status
NCT02481323	Lacunar intervention trial 1 (LACI-1)	Interventional	Isosorbide mononitrate and cilostazol	Completed
NCT00989716	The efficacy of nitric oxide in stroke (ENOS) trial	Interventional	Transdermal glyceryl trinitrate patch	Unknown
NCT03451591	Lacunar Intervention (LACI-2) trial-2	Interventional	Isosorbide Mononitrate, Cilostazol	Completed
NCT01811693	Field administration of stroke therapy-blood pressure lowering	Interventional	Glycerly Trinitrate	Terminated
NCT06715007	Antiplatelet therapy and endothelial-stabilizing agents in cerebral small vessel diseases	Observational	Clopidogrel, Aspirin and Cilostazol + Isosorbide mononitrate	Recruiting
NCT03743103	Esmolol for the treatment of hypertension after intracerebral hemorrhage study (ETHICHS)	Interventional	Brevibloc 10 mg/mL intravenous solution and Nitroprusside sodium	Terminated
NCT07044232	Nicardipine for fast achievement of systolic bp targets in ich	Interventional	Nicardipine + Glyceryl trinitrate	Not_yet_recruiting
NCT03023449	Diffuse optical monitoring with inhaled nitric oxide	Interventional	Nitric oxide	Active_not_recruiting
NCT04988932	Inhaled nitric oxide treatment for aneurysmal SAH patients with intractable cerebral vasospasm	Interventional	Inhaled nitric oxide	Completed
NCT05871606	Inhaled nitric oxide in acute ischemic stroke patients undergoing mechanical thrombectomy	Interventional	Inhaled nitric oxide	Not_yet_recruiting
NCT05762146	Networked drug repurposing for mechanism-based neuroprotection in acute ischaemic stroke	Interventional	Riociguat, Propylthiouracil and Perphenazine	Recruiting
NCT02327793	Perfusion and antihypertensive therapy in acute ischemic stroke	Interventional	Nitroglycerin and Labetalol	Completed
NCT00226096	Intensive blood pressure reduction in acute cerebral haemorrhage	Interventional	Glycerol trinitrate, Nicardipine and Nitroprusside	Completed
NCT01996436	The intra-arterial vasospasm trial	Interventional	Nicardipine, Verapamil and Nicardipine + Verapamil + Nitroglycerin	Terminated

SAH: Subarachnoid hemorrhage.

### Approved Drugs Related to Nitric Oxide and Its Donors in Stroke

As of now, no NO-derived agents have received approval from either U.S. Food and Drug Administration or China Food and Drug Administration for use in clinical stroke treatment. Current agents such as glyceryl trinitrate and SNP are only approved for angina or hypertensive crises, and their use in stroke remains investigational. These findings suggest that the clinical translation of NO and its donors remain at an early stage and requires further development.

## Discussion

**Figure 5:**
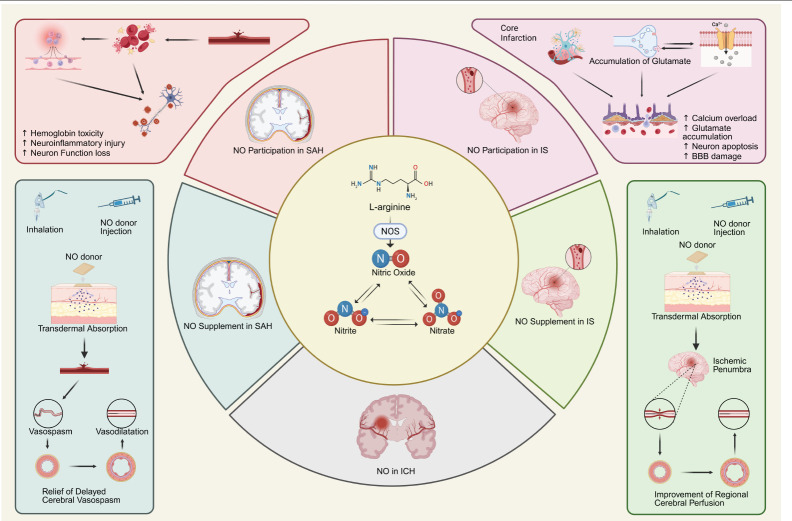
Nitric oxide (NO) in different types of strokes. NO contributes to the pathophysiology of all three major stroke subtypes. In SAH, hemoglobin-mediated NO scavenging triggers neurotoxic cascades and amplifies neuroinflammation, ultimately compromising neurologic function. Timely supplementation with exogenous NO or its donors, however, can attenuate delayed cerebral vasospasm and partially restore cerebral blood flow. In IS, NO, via calcium overload, drives glutamate accumulation within the infarct core, generating excitotoxic injury that leads to neuronal apoptosis and blood–brain barrier disruption. Conversely, exogenous NO enhances perfusion in the ischemic penumbra, thereby salvaging at-risk tissue. By contrast, the role of NO and its donors in ICH remains poorly characterized and warrants further mechanistic and clinical investigation. Created with BioRender.com. BBB: Blood–brain barrier; ICH: intracerebral hemorrhage; IS: ischemic stroke; NOS: nitric oxide synthase; SAH: subarachnoid hemorrhage.

In the preceding section, we summarized the role of exogenous NO supplementation in three different types of strokes, as well as some of the pathophysiological mechanisms involved in these events. **Figure 5** summarily illustrated NO supplementation and pathophysiological process. The vasodilatory effects of NO on cerebral blood vessels have long been recognized by the scientific community, prompting early research into NO donors and their dosages. Currently, the most commonly used NO donors include nitroglycerin, SNP, and sodium nitrite. Nitroglycerin is the most widely applied. Over many years of clinical practice, neurosurgeons have consistently observed that patients with SAH may develop delayed CVS approximately 1 week after the onset of hemorrhage. To reduce secondary brain injury associated with this complication, investigators have initiated the administration of nitroglycerin starting around 1 week after SAH onset. A number of studies have also shown that the use of other NO donors can significantly improve cerebral perfusion and promote neurological recovery in patients with SAH. These findings suggest that NO may exert neuroprotective effects, potentially through mechanisms involving the enhancement of cerebral blood flow. Studies related to IS have also indicated that nitroglycerin is a safe and effective agent for dilating small cerebral blood vessels, significantly alleviating post-stroke vasospasm. Common administration routes include intra-arterial injection, continuous intravenous infusion, and transdermal delivery. In current clinical trials, the most frequently used method is transdermal delivery, typically at a dose of 5 mg/d. Although the duration of administration may vary, this approach has shown improvement in the modified Rankin scale scores of patients at 90 days, suggesting that NO supplementation has the potential to improve post-SAH outcomes. However, studies have also pointed out that nitroglycerin, as an organic NO donor, has the drawback of developing tolerance. In animal experiments, scientists have developed new NO donors and explored new administration routes, such as nasal delivery. In recent years, there has been a significant acceleration in the discovery of innovative drug delivery methods. Particularly noteworthy is the integration of nanoplatforms with ultrasound technology, which facilitates the direct release of NO across the BBB for *in situ* delivery. This advancement has inaugurated a new frontier in the development of NO donors.^121^

Inhaled NO has become a routine therapeutic agent in cardiac vascular surgery and neonatology, primarily administered via inhalation. Following inhalation, NO diffuses into the bloodstream, dilating pulmonary blood vessels, improving blood circulation in the respiratory system, and reducing pulmonary hypertension. After cardiac vascular surgery, inhaled NO has become the standard treatment for lowering pulmonary artery pressure. In neonatology, for newborns with severe respiratory failure, the inhalation of a mixture of NO and oxygen effectively alleviates pulmonary hypertension resulting from respiratory distress.^167^ Compared to other administration methods, inhalation of NO is more straightforward, particularly for intubated patients, making the procedure simpler and more convenient. This clinical practice raises questions about whether inhaled NO may also be applicable to cerebrovascular diseases. Currently, only a few studies have investigated this issue. For instance, a clinical trial by Fung et al.^168^ in 2022 indicated that inhaled NO effectively improved blood flow in small vessels of patients with SAH, with an upper inhalation concentration limit of 40 ppm, but without a clear standard. Research on inhaled NO in cerebrovascular diseases remains limited; therefore, there is a need for more large-scale, multi-center clinical trials to further explore its role in stroke.

With increasing mechanistic insight, the prevailing view of NO in cerebral ischemia has shifted from a purely vaso-protective mediator to a molecule exerting dual effects, encompassing both neuroprotective and neurotoxic actions. Experimental and clinical studies consistently demonstrate that administration of NO donors prior to successful vascular recanalization paradoxically enlarges infarct volume and worsens neurological outcome, a finding that directly contradicts the original therapeutic rationale of improving collateral perfusion. Prolonged ischemia triggers an endogenous surge in NO production. Within minutes, compensatory activation of eNOS and nNOS isoforms generates supraphysiological NO concentrations that precipitate synaptic glutamate accumulation. Excess glutamate overstimulates NMDAR, driving massive Ca²^+^ influx that allosterically amplifies NOS activity and perpetuates a self-reinforcing cytotoxic cascade.^106^,^169^ Parallel to this neuronal compartment, ischemic parenchyma activates resident microglia, polarizing them toward a pro-inflammatory phenotype characterized by robust iNOS expression and sustained NO release. Recent single-cell transcriptomic evidence further reveals that inflammatory cytokines similarly up-regulate iNOS in reactive astrocytes, thereby amplifying local NO load.^170^ The resultant nitrosative stress increases peroxynitrite formation, exacerbates neuronal cytotoxic edema, and disrupts BBB integrity, culminating in secondary expansion of ischemic injury. Although neuroinflammation is indispensable for the clearance of necrotic debris, excessive NO-driven inflammatory amplification becomes maladaptive and inflicts additional neuronal loss. Nevertheless, NO-mediated vasodilation within the ischemic penumbra remains functionally relevant, enhancing microvascular perfusion as long as macrovascular recanalization is achieved. Consequently, the overall impact of NO in acute IS is strictly context-dependent: detrimental when recanalization is absent, yet potentially beneficial following successful vessel reopening.

The aforementioned studies predominantly concentrated on the direct supplementation of NO or NO donors to augment local NO levels and bioavailability. Investigation in 2003 demonstrated that mice deficient in eNOS and nNOS exhibited no protective effects against cerebral ischemic preconditioning, thereby reinforcing the critical role of NO in post-ischemic protection.^24^ Atochin et al.^171^ has proposed that the level of eNOS phosphorylation was closely correlated with IS area. Furthermore, Li et al.^172^ confirmed that defective eNOS phosphorylation can result in an increased stroke area. Additionally, Yemişçi et al.^173^ identified that the 27-bp repeat polymorphism in intron 4 of the NOS gene confers protection against lacunar cerebral infarction. With the advancement of scientific understanding regarding the mechanisms of NOS, researchers have since the 2010s begun investigating the use of peptides or pharmacological agents that modulate NOS activity to mitigate vasospasm following IS or SAH. In 2016, Munakata et al.^174^ reported that oral administration of celecoxib in a rabbit model of SAH elevated eNOS levels, thereby enhancing local NO production and effectively alleviating CVS. Similarly, in 2018, Sun et al.^175^ demonstrated that intraperitoneal injection of Salvinorin A, a peptide, in SAH mice increased eNOS expression and local NO concentrations, contributing to the amelioration of vasospasm. Furthermore, isoflurane, a sedative anesthetic gas, was shown to alleviate delayed CVS post-SAH by downregulating iNOS expression.^176^ In the context of IS research, Yang et al.^177^ found that intravenous administration of the synthetic peptide Adropin significantly reduced the infarct size in mice subjected to MCAO, with its protective effects closely associated with elevated eNOS expression. In recent years, there has been extensive research on drugs targeting the nNOS-PSD95 pathway. Notably, ZL006, a small molecule that disrupts the interaction between nNOS and PSD95, has demonstrated significant neuroprotective effects in both *in vitro* and *in vivo* models. This compound effectively reduces ischemic damage by selectively inhibiting the formation of the nNOS-PSD95 complex, thereby mitigating neuronal apoptosis and enhancing functional recovery. Furthermore, other potential uncouplers of the nNOS-PSD95 interaction, such as coptisine, chelerythrine, and nitidine chloride, have been identified and shown to exert neuroprotective effects in preclinical studies.^178^ Additionally, statin has been identified as an inhibitor of the nNOS-PSD95 pathway,^179^,^180^,^181^,^182^ which is associated with improved local cerebral perfusion and sustained neuroprotective effects. In 2024, Yokomizo et al.^183^ demonstrated that pretreatment of MCAO mice with a 1064 nm laser at an irradiance of 50 mW/cm² significantly enhanced cerebral blood flow, eNOS phosphorylation levels, and stroke prognosis in the mice. These findings strongly suggest that the development of novel nano-platforms could represent a promising strategy to interfere with the NOS pathway, thereby enhancing cerebral blood flow and alleviating brain edema following stroke.

Clinicians should pay special attention to the timing of endogenous NO in the pathophysiological mechanisms during the occurrence of the three different types of strokes. Consequently, the optimal timing for intervention varies significantly across the three major stroke subtypes. In SAH, the concentration of NO in the brain undergoes dynamic changes characterized by a sharp decline, recovery to baseline levels, subsequent excessive generation, and eventual return to baseline. Previous research has predominantly focused on alleviating delayed CVS after SAH, which explains why the majority of animal experiments administered treatments approximately 7 days after SAH onset. Based on the observed temporal patterns of NO fluctuations in the brain, early administration of NO-related drugs or donors may confer additional therapeutic benefits to patients with SAH. However, this hypothesis requires validation through further clinical trials or large-scale RCTs. Similar alterations in NO dynamics are also evident in patients with ICH. Nevertheless, due to the distinct pathophysiological mechanisms underlying ICH, the role of NO appears to be more complex. Therefore, additional studies are necessary to determine whether the administration of NO-modulating agents can provide clinical benefits in ICH patients and to establish the optimal timing of intervention, thereby minimizing secondary injury caused by excessive NO elevation. While in the early stages of IS, a rapid and substantial generation of NO occurs in the brain, which not only causes oxidative damage to the affected brain tissue but also exacerbates the accumulation of glutamate following ischemia, further aggravating brain injury. Therefore, in the management of IS, the early and rapid administration of NO and its donors while blood vessels remain occluded is not considered an appropriate therapeutic strategy. However, with the continuous improvement of stroke triage systems, the likelihood of achieving vascular recanalization within the therapeutic time window has significantly increased compared to previous years. Current evidence suggests that the administration of nitroglycerin following vascular recanalization can effectively enhance cerebral blood flow in the ischemic penumbra, improve regional cerebral perfusion, and ultimately lead to better clinical outcomes. These findings indicate that NO supplementation may serve as a viable therapeutic approach to improving cerebral perfusion in IS patients who have undergone vascular recanalization.

Current evidence indicates that, in SAH, administration of NO or NO-derived agents within the ultra-early phase (≤ 6 hours) may confer neurovascular protection. By contrast, administration between 6 hours and 7 days is associated with exacerbated neuronal injury. After 7 days, NO-based therapy reliably ameliorates delayed CVS. In acute IS, early post-recanalization administration of NO donors enhances cerebral perfusion and synergistically salvages the ischemic penumbra. In ICH, NO-modulating agents may assist acute blood-pressure reduction and limit hematoma expansion; however, additional benefits remain to be rigorously validated. Therefore, future research should emphasize analyzing the timing of medication in various stroke types and the specific timing of administration of NO or its donors in order to promote the neuroprotection process and finally improve the prognosis of stroke patients.

The bias assessment underscores methodological limitations in animal studies, particularly in relation to randomization, blinding, and allocation concealment, aligning with previous reports on bias risks in preclinical research. These limitations may undermine the reliability of results. The absence of blinding may arise from practical challenges inherent in animal experiments, such as the difficulty in masking interventions. However, enhancements in randomization and allocation concealment are achievable and should be prioritized. The low risk of selective reporting bias indicates a satisfactory level of result completeness. Nevertheless, caution is advised due to the variability in bias risks across different studies. Future research should rigorously adhere to SYRCLE guidelines to improve methodological transparency and reporting quality, thereby enhancing the validity and generalizability of findings. The ROB2 assessment identified methodological limitations that affect the validity of our synthesis. Deficiencies in randomization suggest inadequate allocation concealment, which may lead to inflated estimates of treatment effects. Deviations from intervention protocols indicate non-adherence to study protocols, while risks of selective reporting raise concerns about potential outcome cherry-picking. While the majority of IS trials exhibited rigorous outcome measurement, the prevalence of concerns necessitates a cautious interpretation of NO efficacy in this context. In the case of SAH, the evidence base is notably limited, with only two studies available. Importantly, no RCTs assessing the use of NO in ICH met the inclusion criteria, highlighting a significant gap in the evidence for this particular stroke subtype.

## Conclusion

NO is primarily produced through the NO_3_^−^-NO_2_^−^-NO pathway and the L-Arg/NOS pathway *in vivo*. By exogenously supplementing NO or modulating NOS expression, local NO concentrations are elevated, enhancing its bioavailability. Current evidence indicates distinct temporal therapeutic windows for NO-based interventions across stroke subtypes. In SAH, ultra-early administration (≤ 6 hours after stroke onset) may confer neurovascular protection, whereas delivery between 6 hours and 7 days risks exacerbating neuronal injury. Beyond 7 days, NO donors effectively ameliorate delayed CVS. In acute IS, early post-recanalization administration of NO enhances cerebral perfusion and promotes salvage of the ischemic penumbra. For ICH, NO-modulating agents show potential in acute blood-pressure reduction and limiting hematoma expansion, though additional benefits require further validation. Overall, NO demonstrates considerable therapeutic potential in both hemorrhagic and IS, yet its efficacy is critically dependent on timing and context of administration. Future research should prioritize elucidating optimal treatment windows and delivery strategies for NO donors in each stroke subtype, particularly through well-designed clinical trials aimed at maximizing neuroprotection and improving long-term functional outcomes.

## Additional files:

***Additional Figure 1:*** *Risk of bias assessment for SAH animal experiments using SYRCLE’s animal experiment risk assessment tool.*

Additional Figure 1 Risk of bias assessment for subarachnoid hemorrhage animal experiments using SYRCLEs animal experiment risk assessment tool.
(A) Detailed risk of bias judgments for each item. (B) Summary of risk of bias. SYRCLE: SYstematic Review Centre for Laboratory animal Experimentation.

***Additional Figure 2:*** *Risk of bias assessment for ischemia stroke animal experiments using SYRCLE’s animal experiment risk assessment tool.*

Additional Figure 2 Risk of bias assessment for ischemia stroke animal experiments using SYRCLEs animal experiment risk assessment tool.
(A) Detailed risk of bias judgments for each item. (B) Summary of risk of bias. SYRCLE: SYstematic Review Centre for Laboratory animal Experimentation.

***Additional Figure 3:*** *Risk of bias assessment for ischemia stroke RCTs using the ROB2 tool.*

Additional Figure 3 Risk of bias assessment for ischemia stroke RCTs using the ROB2 tool.
D1: Bias due to randomization; D2: bias due to deviations from intended intervention; D3: bias due to missing data; D4: bias due to outcome measurement; D5: bias due to selection of reported result; RCT: randomized controlled trial.

***Additional Figure 4:*** *Risk of bias assessment for subarachnoid hemorrhage RCTs using the ROB2 tool.*

Additional Figure 4 Risk of bias assessment for subarachnoid hemorrhage RCTs using the ROB2 tool.
D1: Bias due to randomization; D2: bias due to deviations from intended intervention; D3: bias due to missing data; D4: bias due to outcome measurement; D5: bias due to selection of reported result; RCT: randomized controlled trial.

***Additional Figure 5:*** *Risk of bias assessment for intracerebral hemorrhage RCTs using the ROB2 tool.*

Additional Figure 5 Risk of bias assessment for intracerebral hemorrhage RCTs using the ROB2 tool.
D1: Bias due to randomization; D2: bias due to deviations from intended intervention; D3: bias due to missing data; D4: bias due to outcome measurement; D5: bias due to selection of reported result; RCT: randomized controlled trial.

***Additional Figure 6:*** *Risk of bias assessment for ischemia stroke non-randomized controlled studies using the ROBINS-I tool.*

Additional Figure 6 Risk of bias assessment for ischemia stroke non-randomized controlled studies using the ROBINS-I tool.
D1: Bias due to confounding; D2. bias due to selection of participants; D3. bias in classification of interventions; D4: bias due to deviations from intended interventions; D5: bias due to missing data; D6: bias in measurement of outcomes; D7: bias in selection of the reported result.

***Additional Table 1:*** *Registered clinical research related to nitric oxide and its donors in stroke.*

***Additional file 1:*** *Detailed search strategies.*

Additional file 1 Detailed search strategies

## Data Availability

*All data relevant to the study are included in the article or uploaded as additional files.*
