# Supplementary material for: Therapeutic potential of nitric oxide and its donors in hemorrhagic and ischemic stroke: a systematic review
Source: Med Gas Res. 2026 Jan 6;16(3):241–57. doi: 10.4103/mgr.MEDGASRES-D-25-00161 (PMC12935127; doi:10.4103/mgr.MEDGASRES-D-25-00161)
Supplement: Supplementary file 1 [file MGR-16-241_Suppl1.pdf]

Additional file 1 Detailed search strategies

Retrieval formula for nitric oxide in subarachnoid hemorrhage

PubMed

("Nitric Oxide"[Title/Abstract]) AND ("Subarachnoid Hemorrhage"[Title/Abstract] OR "SAH"[Title/Abstract] OR "Subarachnoid haemorrhage"[Title/Abstract])) AND ("Therapy"[Title/Abstract] OR "Treatment"[Title/Abstract] OR "Therapeutic"[Title/Abstract]) NOT (review [Title/Abstract])) NOT (meta-analysis [Title/Abstract]) NOT (clinical report [Title/Abstract]) NOT (case report[Title/Abstract])NOT (clinical trial protocol[Title/Abstract]) AND (English[Language])

ScienceDirect

("Nitric Oxide"[Title/Abstract]) AND ("Subarachnoid Hemorrhage"[Title/Abstract] OR "SAH"[Title/Abstract] OR "Subarachnoid haemorrhage"[Title/Abstract])) AND ("Therapy"[Title/Abstract] OR "Treatment"[Title/Abstract] OR "Therapeutic"[Title/Abstract]) AND (research articles) AND (English)

Cochrane Library

("Nitric Oxide"[Title/Abstract]) AND ("Subarachnoid Hemorrhage"[Title/Abstract] OR "SAH"[Title/Abstract] OR "Subarachnoid haemorrhage"[Title/Abstract]) AND ("Therapy"[Title/Abstract] OR "Treatment"[Title/Abstract] OR "Therapeutic"[Title/Abstract]) NOT (review) NOT (meta-analysis) NOT (clinical report) NOT (case report) NOT (clinical trial protocol) AND (English [Language])

Retrieval formula for nitric oxide in intracerebral hemorrhage

PubMed

("Nitric Oxide"[Title/Abstract]) AND ("Intracerebral Hemorrhage"[Title/Abstract] OR "ICH"[Title/Abstract] OR " Intracerebral Haemorrhage"[Title/Abstract])) AND ("Therapy"[Title/Abstract] OR "Treatment"[Title/Abstract] OR "Therapeutic"[Title/Abstract]) NOT (review [Title/Abstract])) NOT (meta-analysis [Title/Abstract]) NOT (clinical report[Title/Abstract]) NOT (case report[Title/Abstract])NOT (clinical trial protocol[Title/Abstract]) AND (English[Language])

ScienceDirect

("Nitric Oxide"[Title/Abstract]) AND ("Intracerebral Hemorrhage"[Title/Abstract] OR "ICH"[Title/Abstract] OR " Intracerebral Haemorrhage"[Title/Abstract]) AND ("Therapy"[Title/Abstract] OR "Treatment"[Title/Abstract] OR "Therapeutic"[Title/Abstract]) AND (research articles) AND (English)

Cochrane Library

("Nitric Oxide"[Title/Abstract]) AND ("Intracerebral Hemorrhage" OR "ICH" OR " Intracerebral Haemorrhage") AND ("Therapy"[Title/Abstract] OR "Treatment"[Title/Abstract] OR "Therapeutic"[Title/Abstract])) NOT (review) NOT (meta-analysis) NOT (clinical report) NOT (case report) NOT (clinical trial protocol) AND (English [Language])

Retrieval formula for nitric oxide in ischemia stroke

PubMed

("Nitric Oxide"[Title/Abstract]) AND ("Ischemia stroke"[Title/Abstract] OR "acute ischemia stroke"[Title/Abstract] OR "AIS"[Title/Abstract] OR "stroke"[Title/Abstract] OR "ischemic stroke"[Title/Abstract])) AND ("Therapy"[Title/Abstract] OR "Treatment"[Title/Abstract] OR "Therapeutic"[Title/Abstract]) NOT (review[Title/Abstract]) NOT (meta-analysis[Title/Abstract]) NOT (clinical report[Title/Abstract]) NOT (case report[Title/Abstract]) NOT (clinical trial protocol[Title/Abstract]) AND (English[Language])

ScienceDirect

("Nitric Oxide"[Title/Abstract]) AND ("Ischemia stroke"[Title/Abstract] OR "acute ischemia stroke"[Title/Abstract] OR "AIS"[Title/Abstract] OR "stroke"[Title/Abstract] OR "ischemic stroke"[Title/Abstract])) AND ("Therapy"[Title/Abstract] OR "Treatment"[Title/Abstract] OR "Therapeutic"[Title/Abstract]) AND (research article) AND (English)

Cochrane Library (Title Abstract Keywords)

("Nitric Oxide"[Title/Abstract]) AND ("Ischemia stroke"[Title/Abstract] OR "acute ischemia stroke"[Title/Abstract] OR "AIS"[Title/Abstract] OR "stroke"[Title/Abstract] OR "ischemic stroke"[Title/Abstract])) AND ("Therapy"[Title/Abstract] OR "Treatment"[Title/Abstract] OR "Therapeutic"[Title/Abstract]) NOT (review) NOT (meta-analysis) NOT (clinical report) NOT (case report) NOT (clinical trial protocol) AND (English [Language])
