# Supplementary figures and images for: Therapeutic potential of nitric oxide and its donors in hemorrhagic and ischemic stroke: a systematic review
Source: Med Gas Res. 2026 Jan 6;16(3):241–57. doi: 10.4103/mgr.MEDGASRES-D-25-00161 (PMC12935127; doi:10.4103/mgr.MEDGASRES-D-25-00161)

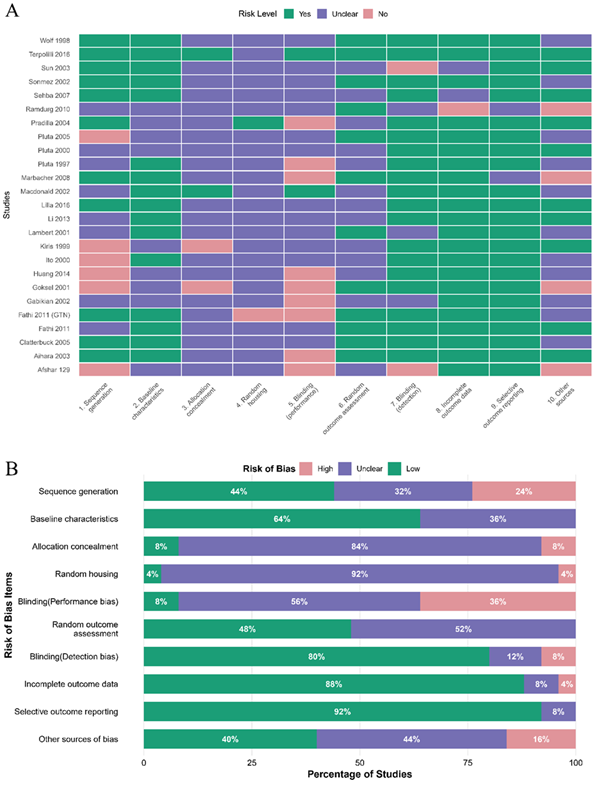

Supplement: Supplementary file 2 [file MGR-16-241_Suppl1.tif]

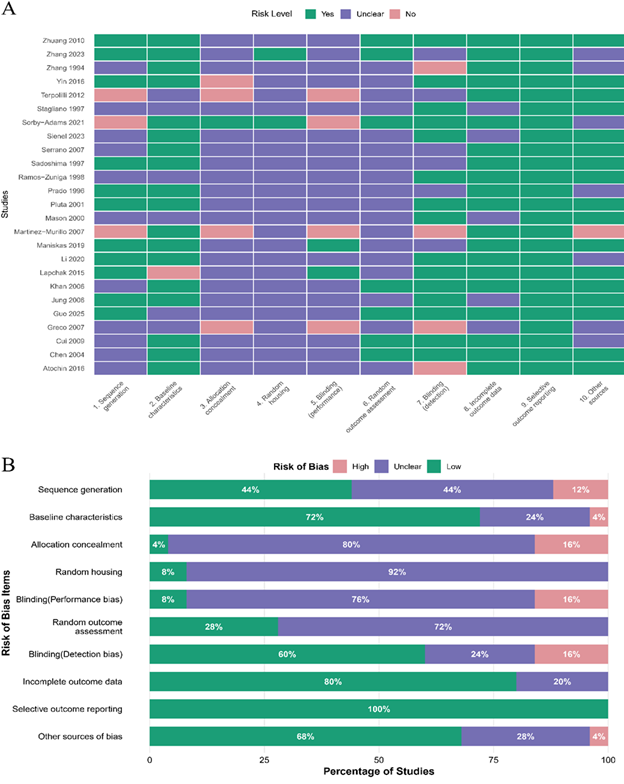

Supplement: Supplementary file 3 [file MGR-16-241_Suppl2.tif]

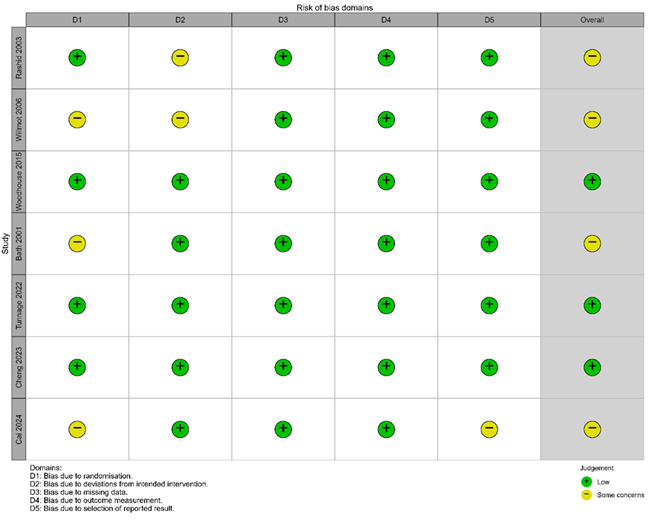

Supplement: Supplementary file 4 [file MGR-16-241_Suppl3.tif]

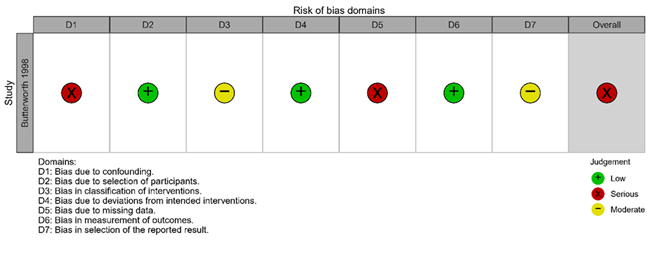

Supplement: Supplementary file 5 [file MGR-16-241_Suppl6.tif]

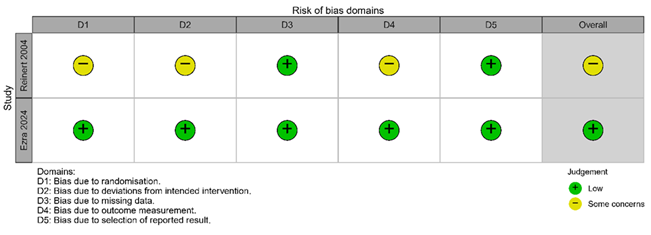

Supplement: Supplementary file 6 [file MGR-16-241_Suppl4.tif]

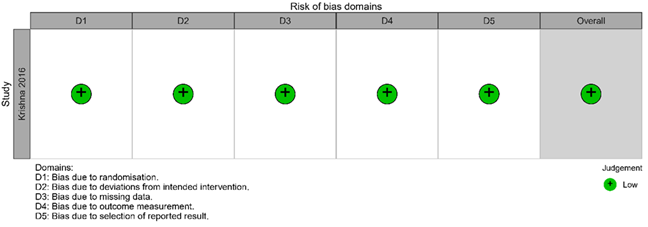

Supplement: Supplementary file 7 [file MGR-16-241_Suppl5.tif]
